# Antiproliferative and biochemical evaluation of rose extracts: impact on tumor and normal skin cells

**DOI:** 10.3389/fpls.2024.1477243

**Published:** 2024-10-30

**Authors:** Cosmin-Alin Faur, Marius Zăhan, Claudiu Ioan Bunea, Eugenia Hârșan, Florin-Dumitru Bora, Andrea Bunea

**Affiliations:** ^1^ Faculty of Animal Science and Biotechnologies, University of Agricultural Sciences and Veterinary Medicine of Cluj-Napoca, Cluj-Napoca, Romania; ^2^ Faculty of Horticulture and Business in Rural Development, University of Agricultural Sciences and Veterinary Medicine of Cluj-Napoca, Cluj-Napoca, Romania; ^3^ Horticultural Research Station, University of Agricultural Sciences and Veterinary Medicine of Cluj-Napoca, Cluj-Napoca, Romania

**Keywords:** anthocyanin-rich fraction, phenolic compounds, HPLC-ESI-MS, cytotoxicity, cell lines

## Abstract

Rose petals (*Rosa* L.) are rich sources of phenolic compounds, including anthocyanins. Anthocyanins and anthocyanidins are associated with multiple health benefits due to their antioxidant properties. In this study, eighteen rose cultivars were comparatively analyzed to determine their total polyphenol and flavonoid content, as well as their antioxidant activity using the 2,2-diphenylpicrylhydrazyl (DPPH) radical scavenging method. The extracts were purified using Amberlite XAD-7 and Sephadex LH-20 columns to obtain anthocyanin-rich fractions. Individual anthocyanins were separated and identified using high-performance liquid chromatography coupled with electrospray ionization mass spectrometry (HPLC-ESI-MS). The three cultivars with the highest anthocyanin content were further examined for cytotoxic effects on cell cultures at various extract concentrations (200-1000 µg/mL) using two skin cell lines: a melanoma cell line (A375) and a normal skin cell line (Hs27). The HPLC-MS analysis identified nine different anthocyanin compounds, with the total anthocyanin content in the rose cultivars varying from 12.42 to 331.95 mg of cyanidin-3-glucoside equivalent/100g of fresh weight. The total polyphenol and total flavonoid contents ranged from 289 to 2703 mg gallic acid equivalent/100g fresh weight and 102 to 603 mg catechin equivalent/100g fresh weight, respectively. Antioxidant activity ranged from 450 to 1304 µmol trolox equivalent/g fresh weight. A significant correlation was observed between antioxidant activity and the content of anthocyanins (R = 0.875, *p* < 0.001), flavonoids (R = 0.982, *p* < 0.001), and polyphenols (R = 0.991, *p* < 0.001). Furthermore, principal component analysis, along with dendrograms and heatmaps, illustrated the relationships among these key compounds and their association with antioxidant activity. The MTT assay showed a substantial suppression of A375 cancer skin cells, while simultaneously exhibiting cell proliferation in Hs27 normal skin cells in a concentration-dependent manner. Altogether, results suggest that the anthocyanins from these rose cultivars could be considered as a promising agent for adjuvant treatment of skin melanoma.

## 1 Introduction

Given the abundance of naturally occurring compounds with advantageous biological qualities in their structure, plants in the Rosaceae family are highly valued and utilized in the food, cosmetics and fragrance industries (Mileva et al., 2021). The Rosaceae family exhibits significant variation in vegetative characteristics. Thorns and prickles are common, particularly in genera like *Rosa* and *Rubus*. Inflorescences are primarily monotelic, often taking a branched form. Complex inflorescences, such as the anthela in *Filipendula*, feature branches of varying lengths that can sometimes overshadow the terminal flower. The typical Rosaceae flower has a prominent hypanthium, a cup-like structure encasing the reproductive organs. Fruits exhibit considerable diversity, with follicles considered the ancestral type. Derived fruit forms include achenes, drupes, and pomes. Seeds typically have a thin testa in enclosed fruits, while those from follicles have a thicker protective coat (Kalkman, 2004). One of the key genera in this family is *Rosa*, which includes numerous species known for their diverse uses (Yang and Shin, 2017). They can be cultivated for a variety of purposes, including cut flowers, landscape plants, perfume oils, culinary applications (rose water), and as a source of vitamin C from fruits called hips. There are more than 100 species of *Rosa* in the Northern Hemisphere’s colder and temperate zones. However, most contemporary cultivars are interspecific hybrids derived from only a few of these species: *R. canina*, *R. chinensis*, *R. gallica*, *R. gigantea*, *R. moschata*, *R. multiflora*, *R. phoenicea*, *R. rugosa*, and *R. wichuraina* (Hummer and Janick, 2009).

Over the past decades, research on plant secondary metabolites highlighted their potential to enhance human well-being (Valdés et al., 2015). When faced with the vast array of adverse biotic circumstances, plants evolve a variety of defense mechanisms including the production of phytochemicals (Soliman et al., 2018). Moreover, plant secondary metabolites also represent a significant reservoir of active substances with high pharmaceutical potential (Yue et al., 2016). Polyphenols are among the most extensively researched and widely distributed classes of phytochemical substances (Somani et al., 2015).

Polyphenols include water-soluble pigments, namely anthocyanins. Anthocyanins are widely present in flowers, fruits, vegetables and are responsible for their intense color that varies from red to blue-violet. Higher plants possess these compounds across several tissues, including the petals (Mattioli et al., 2020). Along with other polyphenols and flavonoids, anthocyanins exhibit a broad spectrum of biological activities in the management of certain disorders such as cancer, cardiovascular diseases, diabetes, obesity, and cognitive decline (He and Giusti, 2010; Pojer et al., 2013). Furthermore, anthocyanin compounds possess the capacity to serve as free radical scavengers against detrimental oxidants such as reactive oxygen and nitrogen species (Ali et al., 2016). The strong correlation between antioxidant activity and anthocyanin content has been demonstrated in blueberries (*Vaccinium corymbosum* L) (Bunea et al., 2013), blackberries (*Rubus fruticosus* L.) (Sariburun et al., 2010), elderberry (*Sambucus canadensis* L.) (Ozgen et al., 2010), sour cherry (*Prunus cerasus* L.) juice (Damar and Ekşi, 2012), colored maize (*Zea mays* L.) kernels (Žilić et al., 2012), and also in roses (Choi et al., 2015; Kumari et al., 2017, Kumari et al., 2022). Yang and Shin (2017) have identified some edible rose cultivars with high antioxidant activities which have been significantly correlated with the increased levels of antioxidant compounds such as anthocyanin, flavonoids and phenolics. Ge and Ma (2013) have found a positive correlation between the anthocyanin concentration and the DPPH radical scavenging rate in Yunnan edible rose (*An ning*). The diversity and levels of anthocyanins in roses are influenced by numerous physiological, environmental, genetic and agronomic factors (Lopes-da-Silva et al., 2007).

Several signaling pathways related to the cell cycle are modulated by anthocyanins, used as part of the treatment for mutagenesis and cancer (Fakhri et al., 2020). For instance, anthocyanidin and anthocyanin extracts from blueberries (*Vaccinium uliginosum* L.) inhibited metastatic murine melanoma cell proliferation by blocking cell cycle progression, thus inducing apoptosis (Wang et al., 2017). Another study indicated that anthocyanins from elderberries could reduce melanoma proliferation and induce apoptosis (Rugină et al., 2017). Moreover, anthocyanins isolated from fruits of *Vitis coignetiae* Pulliat can suppress cancer migration and proliferation in lung cancer cells (Lu et al., 2017). Additionally, anthocyanin-rich extract from black carrot concentrate inhibited the proliferation of HT-29 and HL-60 cell lines (Netzel et al., 2007). Up to now, the antiproliferative activity of rose petals has only been discussed in a small number of studies. For instance, Zu et al. (2010) investigated cytotoxic activities of ten essential oils on three human tumor cell lines (A-549, PC-3 and MCF-7) and concluded that *Rosa damascena* possesses antitumor and anticancerogenic activities. Different concentrations of *Rosa damascena* extracts have been shown to reduce the cellular viability in malignant cells (HeLa) in a concentration- and time-dependent manner (Zamiri-Akhlaghi et al., 2011). Furthermore, an extract from *Rosa x hybrida* petals was shown to stimulate autophagy and apoptosis, which in turn exhibited antiproliferative action in ovarian cancer cells (Rivas-García et al., 2021). Extracts from *Rosa rugosa* petals had a strong antiproliferative effect on HeLa and T47D cancer cell lines (Nowak et al., 2014). Further investigations of different varieties of roses are still important to better understand the potential effects of rose petals on cancer cell lines and the related mechanism.

This study aimed to provide valuable insights into the phytochemical composition including anthocyanins, flavonoids, total polyphenol content, as well as antioxidant activity of eighteen different rose (*Rosa* L.) cultivars and investigate the potential health-related benefits, particularly focusing on antiproliferative activity, which could have implications in medical and pharmaceutical research. The three rose cultivars with the highest anthocyanin content were selected and subjected to detailed analysis using cell culture models to assess and determine their antiproliferative and proliferative activity on A375 tumor cell line and Hs27 normal cell line, respectively. Considering the limited research available on roses, this study offers novel perspectives on their biochemical composition. Based on our current knowledge and available literature, there have been no studies conducted on the effects of roses anthocyanins on A375 and Hs27 cell lines. Moreover, this investigation may strengthen the hypothesis that high antioxidant activity is correlated with the increased level of phenolic content, as well as the linking of anthocyanins with antiproliferative activity in certain types of cancer.

## 2 Materials and methods

### 2.1 Plant material

**Figure 1:**
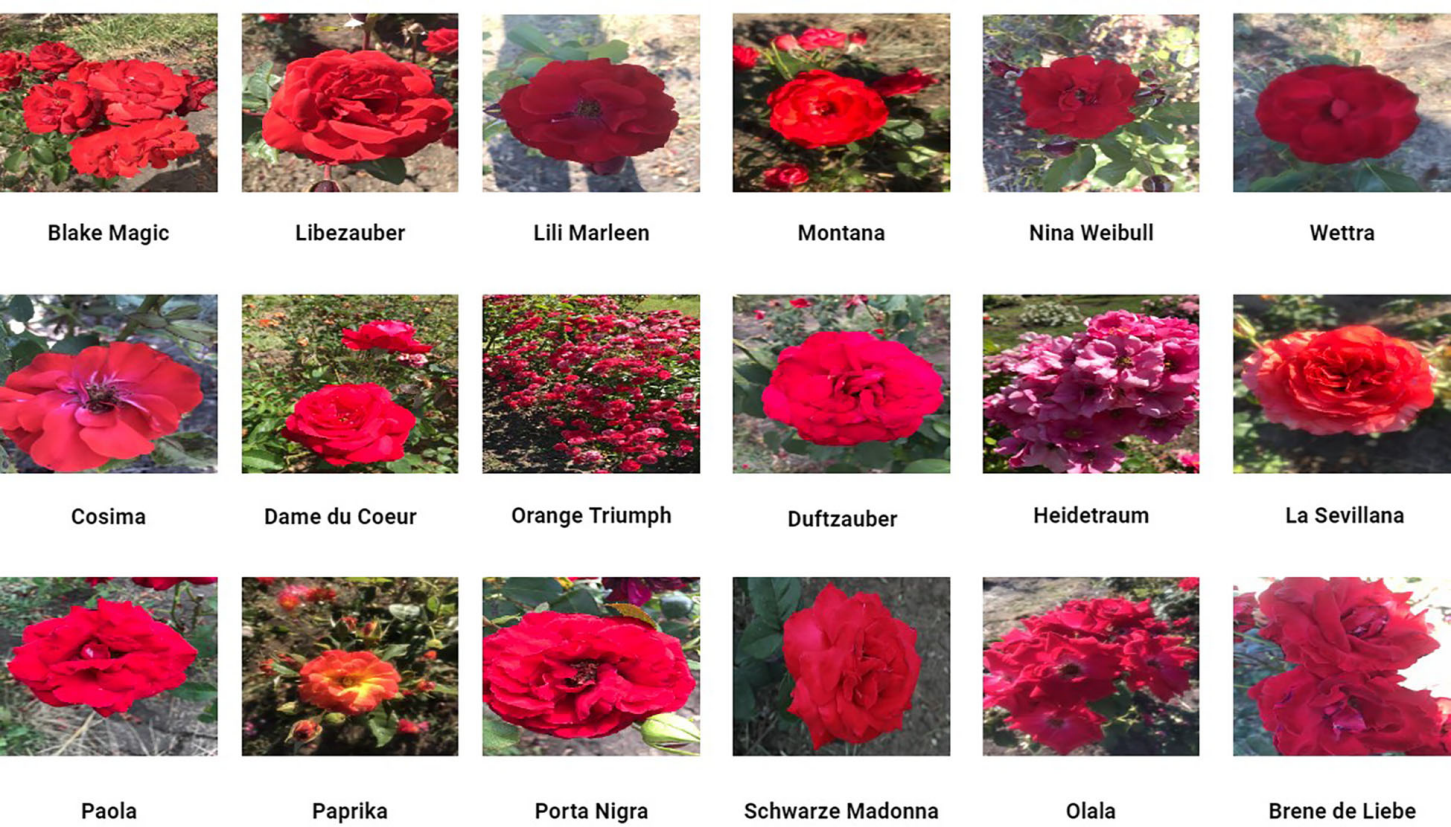
Rose (*Rosa* L.) cultivars analyzed in the study.

Eighteen cultivars of rose (**Figure 1**) were obtained from the Horticultural Research Station Cluj (University of Agricultural Sciences and Veterinary Medicine Cluj-Napoca) in the fall of 2022. The varieties of roses studied are of foreign origin and are part of the domestic collection within Horticultural Research Station (HRS) Cluj-Napoca, which counts approximately 320 varieties (foreign and Romanian). HRS is situated in the northwestern region of Romania (46.758N, 23.618E), with the experimental rose field positioned at an altitude of 369 meters on the northern slope of Feleac Hill. The climate in this area is classified as continental, influenced by the nearby Apuseni Mountains. In the summer, the average temperature is approximately 18°C, though peaks of 35-40°C can occur during heatwaves. Precipitation is relatively low compared to spring and autumn, when precipitation is characterized by more frequent, but less intense rainfall events. In contrast, winters are cold, with temperatures often dropping below freezing, though they rarely fall below -10°C (Salcă Roman et al., 2024). These environmental conditions, along with the region’s fertile soil, provide an ideal environment for the cultivation and study of various rose cultivars. The planting distances varied according to the type of rose, as follows: Hybrid tea roses were planted 90 cm apart between rows and 40 cm apart within rows; Floribunda roses at 90 × 70 cm; Shrubs at 1.5 m × 1.5 m; Ground cover at 2.0 m × 2.0 m; and Climbers at 1.5 m × 1.5 m. The land was kept grassy between the rows, while the rows of bushes were maintained as black fields to aerate the soil and control weeds, which were managed through three to four manual weedings per year to a depth of 8-10 cm. The rose collection was not irrigated, and standard practices such as fertilization, pruning, shoot reduction, winter frost protection, and phytosanitary treatments were implemented (Salcă Roman et al., 2024). After being harvested, the rose plants were transported to the Department of Chemistry and Biochemistry, where the petals were removed and stored in plastic bags in a freezer at -80°C until analysis.

### 2.2 Chemical reagents

Cyanidin-3-glucoside standard was purchased from Polyphenols (Sandnes, Norway). Amberlite XAD-7, Sephadex LH-20, 1,1-Diphenyl-2-picrylhydrazyl (DPPH), gallic acid and catechin standards were purchased from Sigma Chemical Co. (St. Louis, MO, USA). Methanol, trifluoroacetic acid (TFA), ethyl acetate, ethanol, formic acid, acetonitrile and Trolox were purchased from Merck (Darmstadt, Germany). Folin-Ciocalteu’s reagent was purchased from VWR International (Radnor, PA, USA).

Dulbecco’s Modified Eagle Medium was purchased from VWR International (Radnor, PA, USA). Fetal bovine serum was purchased from Biowest (Nuaillé, France). Trypsin powder substance and dimethyl sulfoxide were purchased from Merck (Darmstadt, Germany). Phosphate buffered saline (PBS) tablets (without Ca^2+^ and Mg^2+^) were purchased from MP Biomedicals. Thiazolyl blue tetrazolium bromide powder and glutamine were purchased from Sigma Chemical Co. (St. Louis, MO, USA).

### 2.3 Extraction and determination of anthocyanins

#### 2.3.1 Preparation of anthocyanin fraction

The protocol for extracting anthocyanin fractions from the rose plants has been adapted based on recent studies (Balík et al., 2013; Nile et al., 2015). Anthocyanic and non-anthocyanic compounds were extracted from the petals of the 18 rose cultivars (2g) using acidified methanol (0.3% HCl (v/v)) and a homogenizer (Miccra D-9 KT Digitronic, Bergheim, Germany). The extraction step was repeated until the color of the extraction solvent disappeared, and the final extraction was carried out overnight at 4°C in the dark. Acidified methanol (0.3% HCl) was used to prevent anthocyanin degradation (Nile et al., 2015). The colored extract was filtered through multiple layers of cotton wool and concentrated under vacuum at 35°C until all methanol was removed. To further eliminate polar molecules from the extract, liquid-liquid extraction with ethyl acetate-water was performed. The aqueous fraction was applied to an Amberlite XAD-7 column (1 × 0.5 cm), preconditioned with 6 volumes of 0.3% aqueous TFA. After loading the extract, the column was washed with 4 volumes of 0.3% aqueous TFA to remove sugars, pectins and other free impurities. The anthocyanins and procyanidins were then removed from the column using 4 volumes of methanol (containing 0.3% TFA (v/v)). The organic fraction was then purified on a Sephadex LH-20 column (2.5 × 0.5 cm), pre-conditioned and eluted with 10 volumes of water:methanol (0.3% TFA (v/v)) (8:2), resulting in the anthocyanin-rich fraction. The volume of anthocyanin-rich fraction was adjusted to 5 ml with distilled water, filtered through a 0.45 µm filter and analyzed by High-performance liquid chromatography-diode array detection (HPLC-DAD) and HPLC-electrospray ionization mass spectrometry (HPLC-ESI-MS).

#### 2.3.2 HPLC-DAD analysis of anthocyanins

The analysis was carried out on a Shimadzu HPLC system equipped with an LC-20 AT binary pump system (Prominence), a degasser DGU-20 A3 (Prominence), a set of SPD-M20 diode array detectors, and an ultraviolet-visible (UV-VIS) detector. A Luna Phenomenex C-18 column (5 µm, 25 cm x 4.6 mm) was used. The mobile phase consisted of 4.5% formic acid in distilled water (solvent A) and acetonitrile (solvent B). The gradient elution process started with 10% solvent B for 9 min. The percentage of solvent B was then increased linearly to reach 12% by 17 min and 25% by 30 min. Between 30 and 50 min, the percentage of solvent B was set to 90%. The flow rate was 0.8 mL/min, and the analyses were performed at a temperature of 35°C. Anthocyanin quantification was performed using cyanidin-3-glucoside as a standard in the concentration range of 2.5-500 µg/mL. Chromatograms were recorded at 520 nm wavelength. Total anthocyanins were calculated as the sum of all identified anthocyanins and expressed in mg cyanidin-3-O-glucoside equivalent (CGE)/100g fresh weight (FW).

#### 2.3.3 LC-MS-ESI^+^ analysis

Mass spectrometry (MS) analysis was carried out using an Agilent 1200 HPLC system equipped with a quaternary pump, solvent degasser, autosampler, UV-VIS/DAD and coupled with a single-quadrupole mass detector Agilent model 6110 (Agilent Technologies, CA, USA). Compounds were separated on an Eclipse XDB C18 column (5 μm, 4.6x150 mm, Agilent Technologies, CA, United States), using water + 0.1% acetic acid (solvent C) and acetonitrile + 0.1% acetic acid (solvent D) according to gradient elution at a flow rate of 0.5 ml/min at 250°C for 30 minutes. The gradient (expressed in % D) was as follows: 0 min, 5% D; 0-2 min, 5% D; 2-18 min, 5%-40% D; 18-20 min, 40%-90% D; 20-24 min, 90% D; 24-25 min, 90%-5% D; 25-30 min, 5% D. Spectral values were recorded in the range of 200-600 nm for all peaks. Chromatograms were recorded at wavelengths λ = 280, 340, and 520 nm. For MS, positive electrospray ionization mode was utilized under the following conditions: capillary voltage 3000 V, temperature 3500 C, nitrogen flow rate 7 L/min, nebulization pressure 35 psi, fragmentor voltage 100 eV and m/z range 120-1200 in full-scan mode. Data acquisition and result interpretation were performed using Agilent ChemStation software.

### 2.4 Determination of total flavonoid content

The total flavonoid content was determined using a colorimetric method (Meyers et al., 2003; Shin, 2012). The alcoholic extract was diluted to a final volume of 5 ml with distilled water. After adding 300 μl 5% NaNO_2_ the mixture was allowed to stay 5 min. Then 300 μl AlCl_3_ 10% was added and, after 6 minutes, 2 ml NaOH 1N. The absorbance was measured at a wavelength of 510 nm using a spectrophotometer. Various concentrations of standard catechin (100, 150, 200, and 250 mg/L) were used for the calibration curve. The total flavonoid content was expressed in mg catechin equivalent (CE)/100g fresh weight (FW).

### 2.5 Determination of total phenolic content

The total polyphenol content in the rose extracts was measured using the Folin-Ciocâlteu colorimetric method on a Jasco V-630 UV-Vis spectrophotometer (Meyers et al., 2003; Shin, 2012). Different dilutions of the rose extracts were prepared to ensure that the absorbance readings would fall within the range of the standard calibration curve made with gallic acid (R=0.997). The extracts were oxidized by the Folin-Ciocalteu reagent (120 μl) and the neutralization was made with Na_2_CO_3_ (340 μl), after 5 minutes. The absorbance was measured at 750 nm after 90 minutes in the dark, at room temperature. The results were expressed as mg gallic acid equivalent (GAE)/100g fresh weight.

### 2.6 DPPH radical scavening activity

The antioxidant activity was obtained using a published 2,2-diphenyl-1-picrylhydrazyl (DPPH) method (Sharma et al., 2015) with slight modifications. A stock solution containing 24 mg of DPPH in 100 mL of 75% ethanol was prepared and the solution was stored at -20°C until use. The working solution of DPPH was prepared by diluting the stock solution with 75% ethanol to achieve an absorbance of 1.1 ± 0.02 units at 522 nm. Initially, 1950 µl of the DPPH working solution was mixed with 50 µl of the sample extract, and the mixture was incubated for 20 min. A control experiment was conducted by adding 50 µl of 75% ethanol to the 1950 µl of the DPPH solution. Absorbances were measured in 75% ethanol using a UV/VIS spectrophotometer. The results were expressed in µmol Trolox equivalent (TE) per gram of fresh weight (µmol TE/g FW).

### 2.7 Cytotoxic assay

Among the 18 rose cultivars analyzed, the three richest varieties in anthocyanins including Schwarze Madonna, Lili Marleen, and Porta Nigra were identified. These cultivars were selected for testing the antiproliferative activity on two cell lines at different concentrations The experiments were performed on a human tumor A375 (skin melanoma, ATCC^®^ CRL-1619) cell line and on a normal Hs27 (skin, ATCC^®^ CRL-1634) cell line. Both A375 and Hs27 cell lines were stored in liquid nitrogen (-196°C) at Institute of Life Sciences Cluj-Napoca. Cell lines were grown at 37°C, 5% (v/v) CO_2_ and 95% (v/v) relative humidity in Dulbecco’s Modified Eagle’s Medium containing 2 mM L-glutamine, 1 mM sodium pyruvate and 10% (v/v) fetal bovine serum.

For reconstitution of anthocyanin rich-fractions, 2 mL of methanol was added, and stock solutions of 200 µL were prepared. Working solutions of 200, 400, 600, 800 and 1000 µg/mL FW were then prepared from the stock solutions. About 80% confluence was reached before the cell line was detached using a 0.25% (w/v) trypsin-0.53 mM EDTA solution. The cells were then seeded at a concentration of 5 × 10^4^ per well in 100 µL culture media in 96-well microplates. Following a 24-hour incubation period, the cells were exposed to various concentrations of the extracts (200-1000 µg/mL). Cells in culture media served as the control. The impact of the cytotoxicity assay was evaluated using the 3-(4,5-dimethyl-2-thiazolyl)-2,5-diphenyl-2H-terazolium bromide reagent (MTT). Following the PBS washing step, cells were incubated for 1h at 37°C with a 100 µL/well MTT solution (5 mg/mL). Dimethyl sulfoxide was used to dissolve the resulting formazan crystals at a concentration of 100 µL/well. A microplate reader from BioTek Synergy Instruments (Winooski, VT, USA) was utilized to measure absorbance at 550 and 630 nm. Cell vitality was expressed as a percentage of the control group, which consisted exclusively of cells cultured in normal conditions.

### 2.8 Statistical analysis

**Figure 2:**
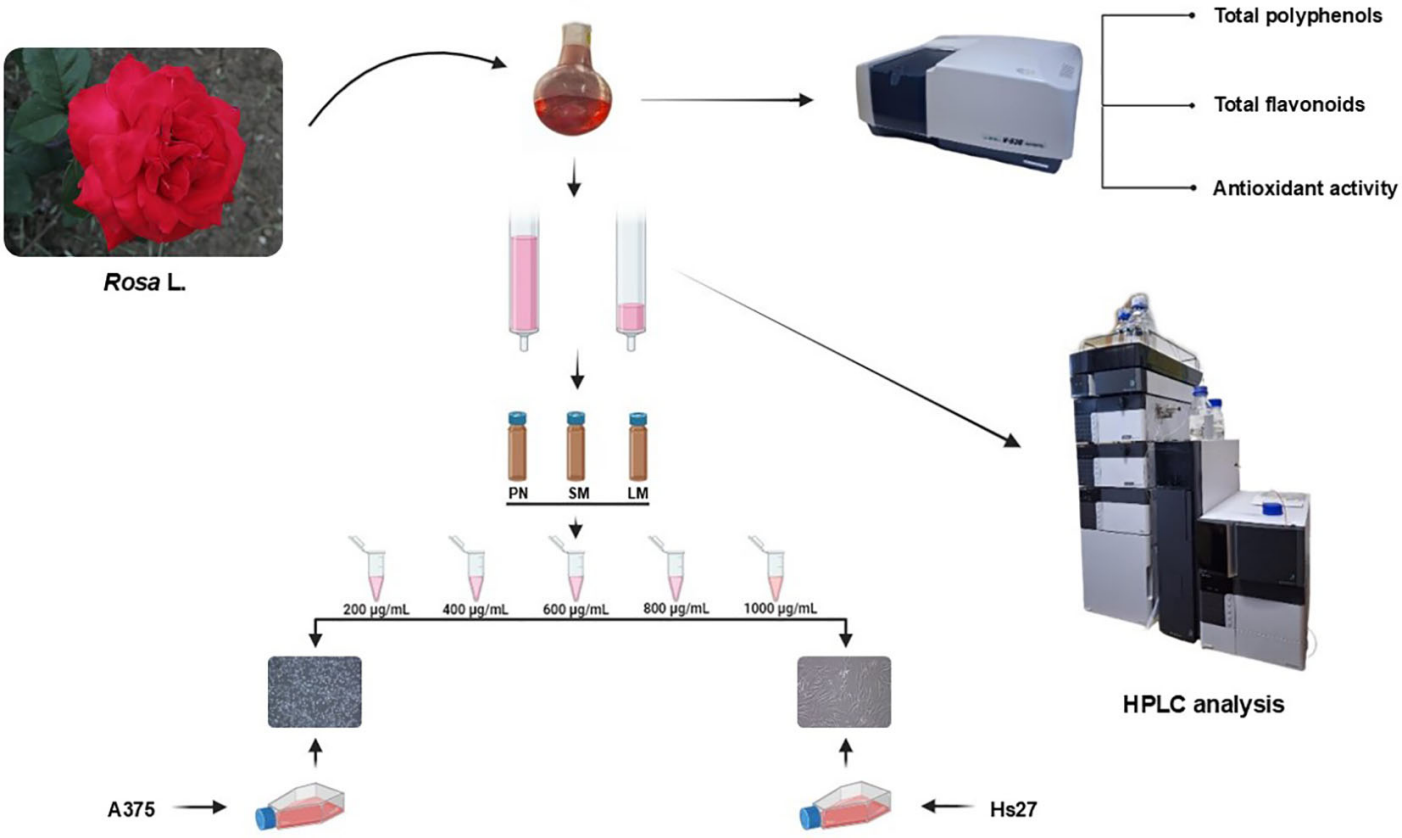
Graphical scheme of workflow procedure.

The data were expressed as mean ± standard deviation (SD) from three replicates for each sample. For the cell culture experiments, one replication means the average of five wells containing anthocyanin rich fraction treated or non-treated (control) cells. Data analysis was performed using a combination of software tools. Microsoft Excel 365 and Addinsoft (2018) were used for initial data exploration, calculating descriptive statistics (averages, medians, standard deviations, and correlations), and exploratory analyses (hierarchical clustering and principal component analysis). The statistical analysis was conducted using IBM SPSS Statistics (Version 26) and GraphPad Prism (Version 8). Compatibility with normal distribution was determined using the Shapiro-Wilk test. One-Way ANOVA and Duncan’s Multiple Range tests (*p* < 0.05) were performed to examine the differences among groups. Correlations were analyzed using Pearson’s correlation coefficient (*p* < 0.001). A graphical representation of the workflow procedure is shown in **Figure 2**.

## 3 Results

### 3.1 Identification and quantification of anthocyanins in rose petals

**Figure 3:**
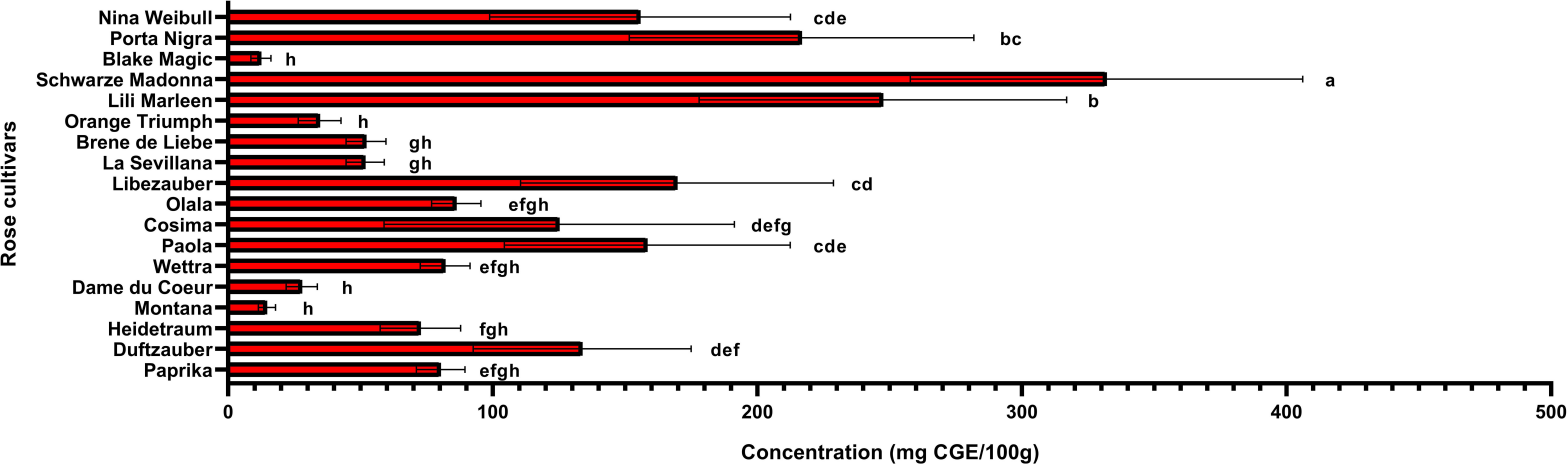
Total anthocyanin concentration in roses (*Rosa* L.). Data expressed as mean values (Measurements carried out in triplicate); error bars depict standard deviations. Values with different letters (a−h) are significantly different (*p* < 0.05), using Duncan’s Multiple Range Test.

**Figure 4:**
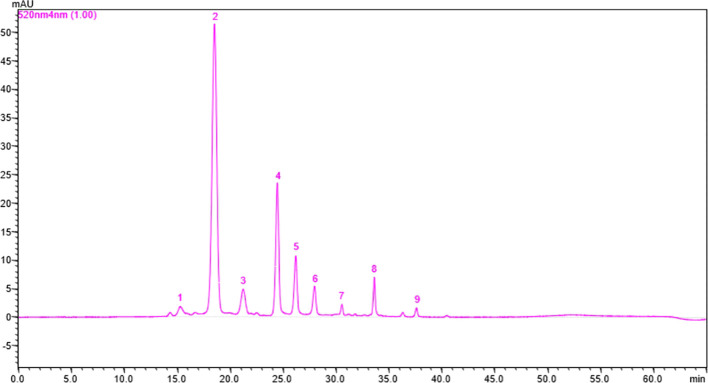
The HPLC chromatogram for anthocyanin separation in Black Magic cultivar. 1 = Delphinidin-rutinoside; 2 = Cyanidin-caffeoyl-glucoside; 3 = Cyanidin-coumaroyl-glucoside; 4 = Cyanidin-glucoside; 5 = Pelargonidin-glucoside; 6 = Cyanidin; 7 = Delphinidin; 8 = Peonidin-arabinoside; 9 = Pelargonidin (Peak numbers correspond with those from **Table 1**). The identification of individual anthocyanins was made based on HPLC-ESI-MS.

Anthocyanin content is an important factor that determines whether roses are red or violet in color and the degree to which they influence antioxidant activity. The total anthocyanin content for each extract obtained from the rose petals was determined by HPLC-DAD analysis (**Figure 3**). The total anthocyanin content varied between 12.42 ± 3.6 mg CGE/100g FW in Black Magic variety and 331.95 ± 74.12 mg CGE/100g FW in Schwarze Madonna variety. High values of anthocyanin content were also identified in the Liebeszauber (169.63 ± 59.1 mg CGE/100g FW), Paola (158.38 ± 54 mg CGE/100g FW) and Nina Weibull (155.62 ± 56.87 mg CGE/100g FW) varieties. The total anthocyanin content in Schwarze Madonna cultivar was significantly higher as compared to that of the other rose cultivars, while there was no significant difference between Porta Nigra and Lili Marleen variety. Moreover, there was no significant difference between Porta Nigra variety and Paola, Libezauber or Nina Weibull cultivars. In total, 9 individual anthocyanins were identified (**Table 1**). A representative example of HPLC profile of the anthocyanin-rich fractions of Black Magic cultivar is given in **Figure 4**. The other chromatograms are presented in **Supplementary File**. Even though Black Magic variety had the lowest concentration in total anthocyanin content, it exhibited all individual anthocyanins alongside Cosima variety. Cyanidin-caffeoyl-glucoside was identified as the most abundant compound in rose petals (**Table 2**). Schwarze Madonna, Lili Marleen and Porta Nigra cultivars exhibited high amounts of this compound, while Montana and Black Magic varieties recorded low concentrations. The levels of delphinidin-rutinoside vary significantly among cultivars, with Porta Nigra and Schwarze Madonna cultivars showing the highest concentrations (8.499 ± 2.14 and 5.91 ± 1.46 mg CGE/100g FW, respectively), while Paprika cultivar recorded lower amounts (2.223 ± 0.28 mg CGE/100g FW). Cosima variety exhibited the highest concentration of cyanidin-coumaroyl-glucoside (31.62 ± 21.28 mg CGE/100g FW). Although the concentrations of cyanidin-glucoside were relatively low, it was present in all rose varieties analyzed. The highest amount of pelargonidin-glucoside was recorded in Libezauber cultivar (12.65 ± 4.89 mg CGE/100g FW). Among the anthocyanidins, cyanidin was the most abundant compound, detected in all cultivars except for the Nina Weibull variety. In contrast, pelargonidin was exclusively detected in Cosima, Black Magic and Montana cultivars. A significant correlation (R=0.875, *p* < 0.001) was found between the overall anthocyanin concentration and their antioxidant activity.

**Table 1:** Retention time, UV-VIS and mass spectral data of anthocyanins in the rose cultivars analyzed.

Peak No.	Rt (min)	UV λ_max_ (nm)	[M+H]+ (*m/z*)	Compound
1	15.33	517, 276	611	Delphinidin-rutinoside
2	18.54	520, 330, 280	611	Cyanidin-caffeoyl-glucoside
3	21.27	520, 320, 280	595	Cyanidin-coumaroyl-glucoside
4	24.50	520, 280	449	Cyanidin-glucoside
5	26.23	520, 270	433	Pelargonidin-glucoside
6	28.01	520, 280	287	Cyanidin
7	30.60	517, 276	303	Delphinidin
8	33.66	520, 274	449	Peonidin-arabinoside
9	37.60	520, 270	271	Pelargonidin

**Table 2:** Individual anthocyanin content in rose cultivars, expressed as mg CGE/100g FW.

Cultivars	Delphinidin-rutinoside	Cyanidin-caffeoyl-glucoside	Cyanidin-coumaroyl-glucoside	Cyanidin-glucoside	Pelargonidin-glucoside	Cyanidin	Delphinidin	Peonidin-arabinoside	Pelargonidin
**Paprika**	2.223 ± 0.28^de^	49.51 ± 6.05^hi^	17.49 ± 1.57^bc^	2.735 ± 0.27^efgh^	4.015 ± 0.55^cd^	1.895 ± 0.19^f^	2.476 ± 0.3^cd^	nd	nd
**Duftzauber**	nd	111.7 ± 29.37^defg^	5.005 ± 3.24^cde^	6.393 ± 3.88^bcde^	nd	10.69 ± 4.71^cd^	nd	nd	nd
**Heidetraum**	3.413 ± 0.88^cd^	53.90 ± 10.14^ghi^	1.715 ± 0.68^e^	3.645 ± 0.81^efgh^	nd	8.987 ± 2.24^cde^	nd	1.045 ± 0.45^b^	nd
**Montana**	nd	6.486 ± 0.96^i^	2.956 ± 0.85^de^	0.943 ± 0.46^h^	1.909 ± 0.49^d^	0.661 ± 0.15^f^	1.255 ± 0.18^cd^	0.256 ± 0.09^c^	0.227 ± 0.03^b^
**Dame Du Coeur**	nd	20.4 ± 3.98^hi^	nd	3.198 ± 0.98^efgh^	nd	3.534 ± 0.78^ef^	nd	0.732 ± 0.15^bc^	nd
**Wettra**	nd	71.74 ± 6.19^fgh^	nd	2.837 ± 1.02^efgh^	nd	5.333 ± 1.98^def^	nd	0.924 ± 0.21^bc^	nd
**Paola**	4.099 ± 0.485^bcd^	135.2 ± 45.38^cde^	nd	5.869 ± 1.915^cdef^	nd	13.21 ± 6.22^c^	nd	nd	nd
**Cosima**	3.696 ± 1.22^cd^	58.75 ± 31.41^ghi^	31.62 ± 21.28^a^	5.028 ± 2.48^defg^	6.997 ± 3.02^b^	4.517 ± 2.11^def^	8.874 ± 4.29^a^	0.973 ± 0.15^bc^	1.17 ± 0.24^a^
**Olala**	nd	81.26 ± 8.17^efgh^	nd	2.503 ± 0.61^efgh^	nd	2.843 ± 0.52^ef^	nd	nd	nd
**Libezauber**	nd	121.7 ± 41.37^cdef^	19.46 ± 5.44^b^	8.437 ± 3.67^bcd^	12.65 ± 4.89^a^	2.93 ± 1.25^ef^	4.452 ± 2.48^b^	nd	nd
**La Sevillana**	nd	27.42 ± 3.99^hi^	16.02 ± 1.88^bcd^	2.092 ± 0.35^fgh^	1.431 ± 0.15^d^	3.291 ± 0.67^ef^	1.566 ± 0.16^cd^	nd	nd
**Brene de Liebe**	nd	47.2 ± 6.58^hi^	nd	1.52 ± 0.33^gh^	nd	3.424 ± 0.59^ef^	nd	nd	nd
**Orange Triumph**	nd	23.98 ± 6.2^hi^	5.222 ± 1.14^cde^	1.784 ± 0.28^gh^	1.002 ± 0.14^d^	1.833 ± 0.25^f^	0.799 ± 0.09^e^	nd	nd
**Lili Marleen**	nd	214.4 ± 60.32^b^	nd	9.161 ± 1.28^bc^	nd	23.93 ± 7.83^b^	nd	nd	nd
**Schwarze Madonna**	5.91 ± 1.46^b^	273.4 ± 58.43^a^	nd	16.08 ± 4.39^a^	nd	33.72 ± 8.96^a^	nd	2.837 ± 0.88^a^	nd
**Black Magic**	0.445 ± 0.11^e^	6.285 ± 2.27^i^	0.722 ± 0.2^e^	2.078 ± 0.61^fgh^	1.011 ± 0.26^d^	0.618 ± 0.11^f^	0.337 ± 0.08^e^	0.592 ± 0.1^bc^	0.334 ± 0.06^b^
**Porta Nigra**	8.499 ± 2.14^a^	176.9 ± 54.86^bc^	8.557 ± 2.11^bcde^	9.744 ± 3.03^b^	nd	12.98 ± 2.98^c^	nd	nd	nd
**Nina Weibull**	4.765 ± 1.23^bc^	145.4 ± 53.78^cd^	nd	5.46 ± 1.86^cdefg^	nd	nd	nd	nd	nd

*nd, not detected. Individual anthocyanins expressed as mean ± standard deviation (measurements carried out in triplicate). In columns, mean values with different letter for each concentration denote significant difference at *p* < 0.05 (Duncan’s Multiple Range Test).

### 3.2 Determination of total phenolic content, total flavonoids and antioxidant activity

**Figure 5:**
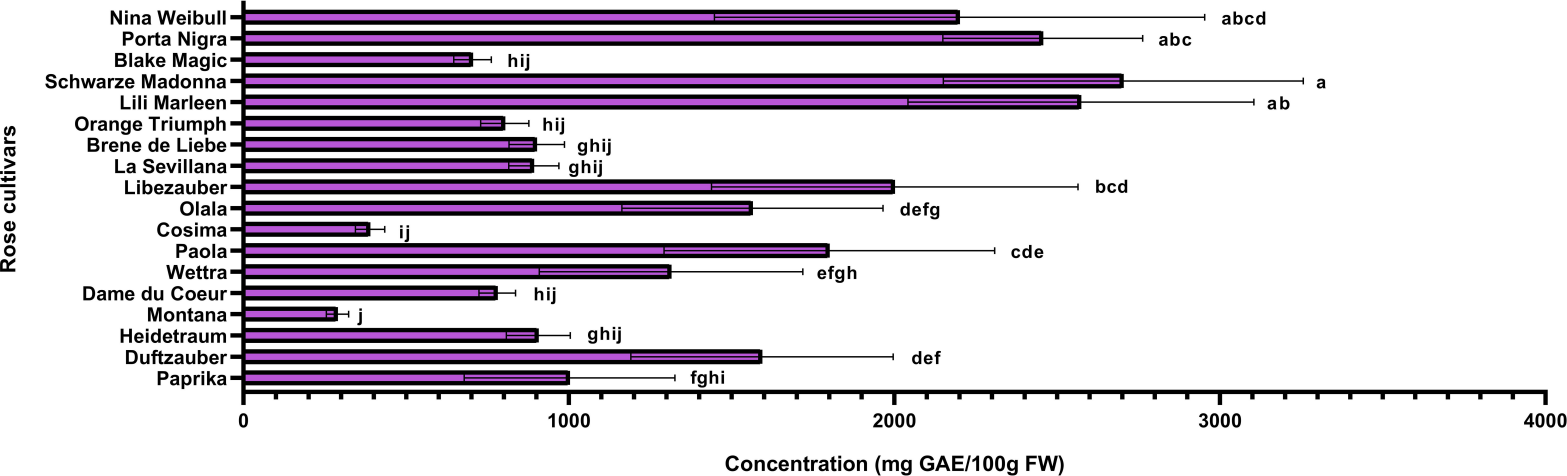
Total polyphenol content in roses (*Rosa* L.). Data expressed as mean values (Measurements carried out in triplicate); error bars depict standard deviations. Values with different letters (a−j) are significantly different (*p* < 0.05), using Duncan’s Multiple Range Test.

**Figure 6:**
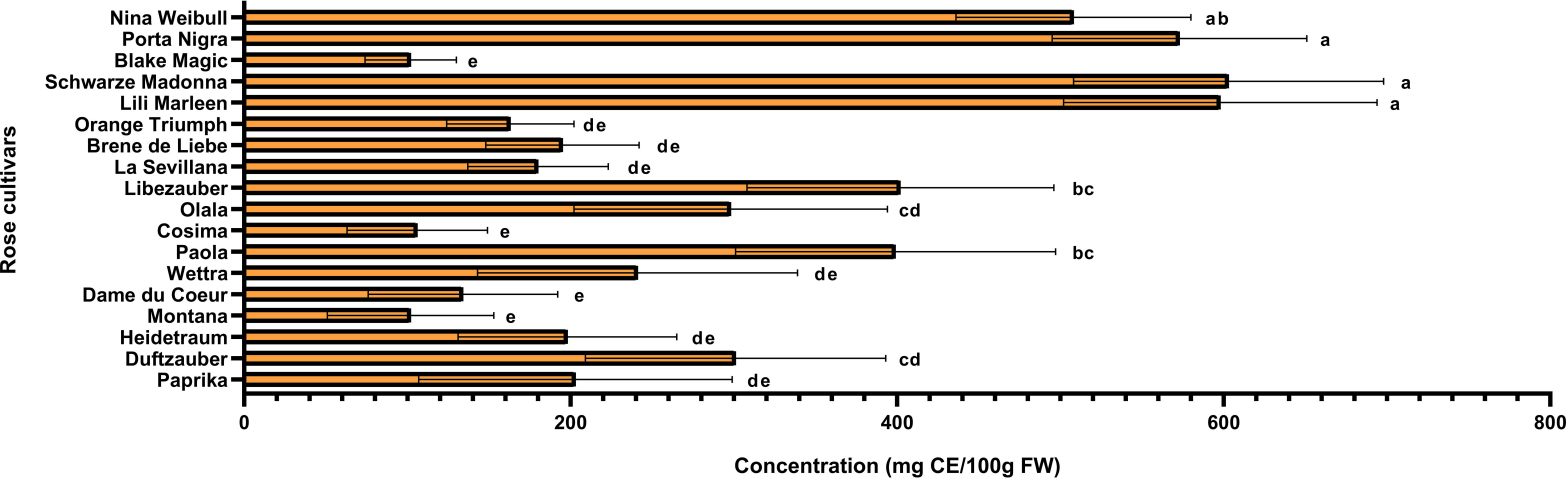
Total flavonoid content in roses (*Rosa* L.). Data expressed as mean values (Measurements carried out in triplicate); error bars depict standard deviations. Values with different letters (a−e) are significantly different (*p* < 0.05), using Duncan’s Multiple Range Test.

**Figure 7:**
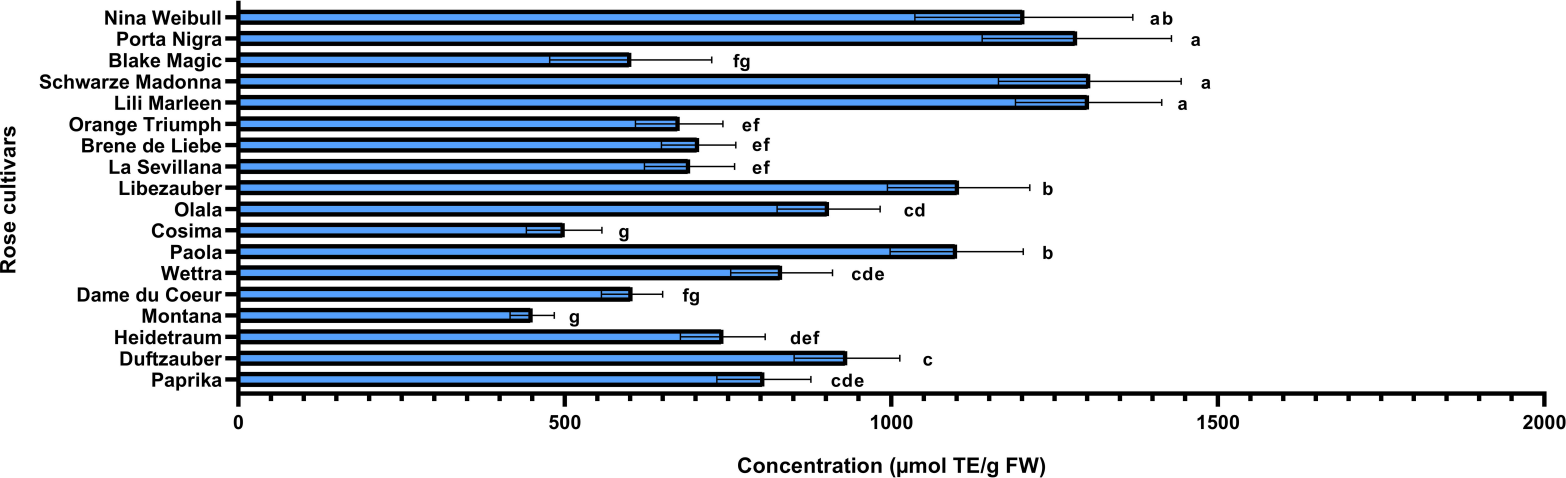
Antioxidant activity in roses (*Rosa* L.). Data expressed as mean values (Measurements carried out in triplicate); error bars depict standard deviations. Values with different letters (a−g) are significantly different (p < 0.05), using Duncan’s Multiple Range Test.

Rose petals, identified as a rich source of polyphenols, were further analyzed to quantify these bioactive compounds and assess the antioxidant capacity of the extracts. The varieties under investigation were evaluated for their total phenolic and flavonoid contents, while their antioxidant activity was assessed using the DPPH radical scavenging assay. Additionally, correlations between total phenolic content, flavonoid levels, and antioxidant activity were examined using Pearson’s coefficient to better understand their relationships. The varieties identified with the highest concentrations of anthocyanins (Schwarze Madonna, Lili Marleen and Porta Nigra) also maintain increased values for total polyphenols, total flavonoids, and antioxidant activity. However, it is important to note that statistical analysis revealed no significant differences in any of these analyses among these varieties. The total polyphenol content ranged from 289 ± 34 mg GAE/100g FW in the Montana variety to 2703 ± 553 mg GAE/100g FW in the Schwarze Madonna variety (**Figure 5**). Additionally, the varieties recorded values for flavonoid content from 102 ± 28 mg CE/100g FW in the Black Magic variety to 603 ± 95 mg CE/100g FW in the Schwarze Madonna variety (**Figure 6**). Furthermore, Montana cultivar exhibited the lowest antioxidant activity value at 450 ± 34 µmol TE/g FW, while the highest value was again observed in the Schwarze Madonna variety at 1304 ± 301 µmol TE/g FW (**Figure 7**). While Lili Marleen, Schwarze Madonna, and Porta Nigra were identified as the richest varieties in phenolics, flavonoids, and antioxidant activity, they showed no significant difference compared to the Nina Weibull variety. Additionally, a strong correlation was found between antioxidant activity and total phenolic content (R=0.991, *p* < 0.001). Also, the correlation between antioxidant activity and total flavonoids was found to be very strong (R=0.982, *p* < 0.001).

### 3.3 The cytotoxic effect of the anthocyanic extract on the A375 and Hs27 cell lines

**Figure 8:**
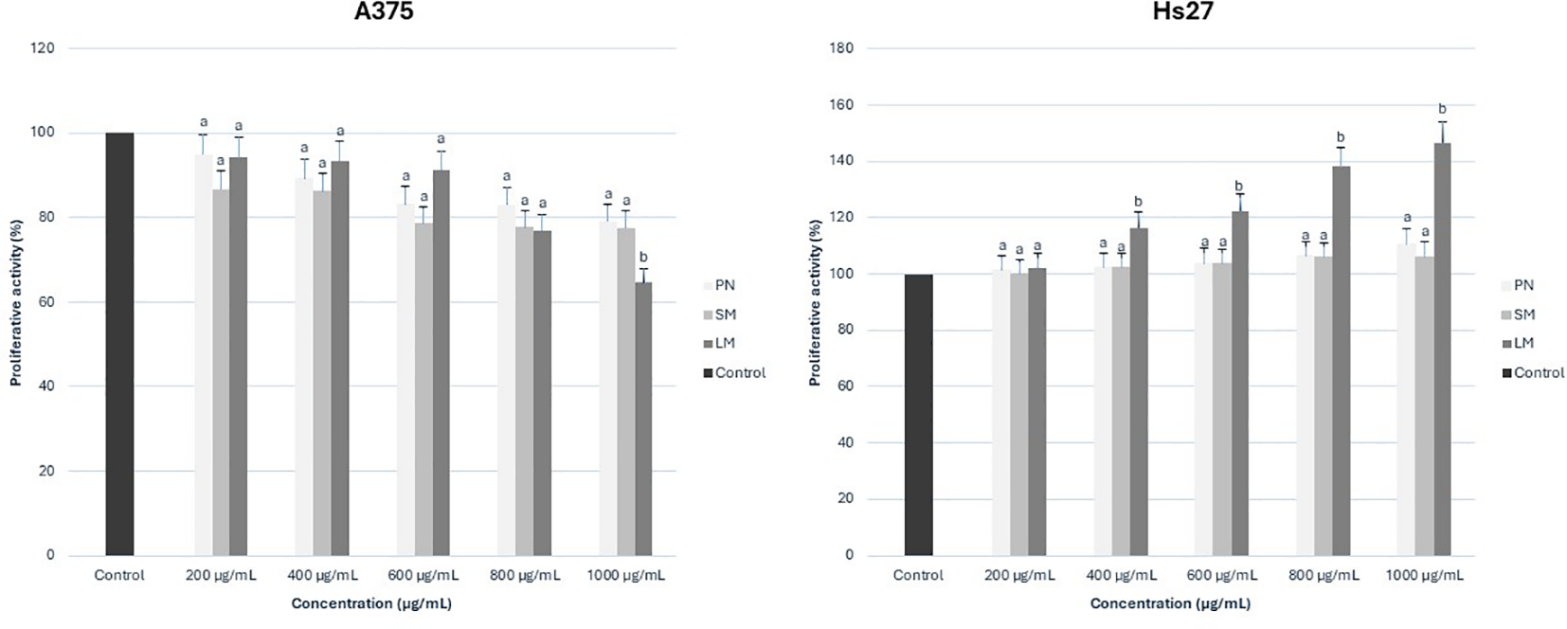
Proliferative activity (on A375 and Hs27 cell lines) of rose (*Rosa* L.) extracts. PN, Porta Nigra cv.; SM, Schwarze Madonna cv.; LM, Lili Marleen cv. Mean values with different letter for each concentration denote significant difference at *p* < 0.05, using Duncan’s Multiple Range Test.

**Figure 9:**
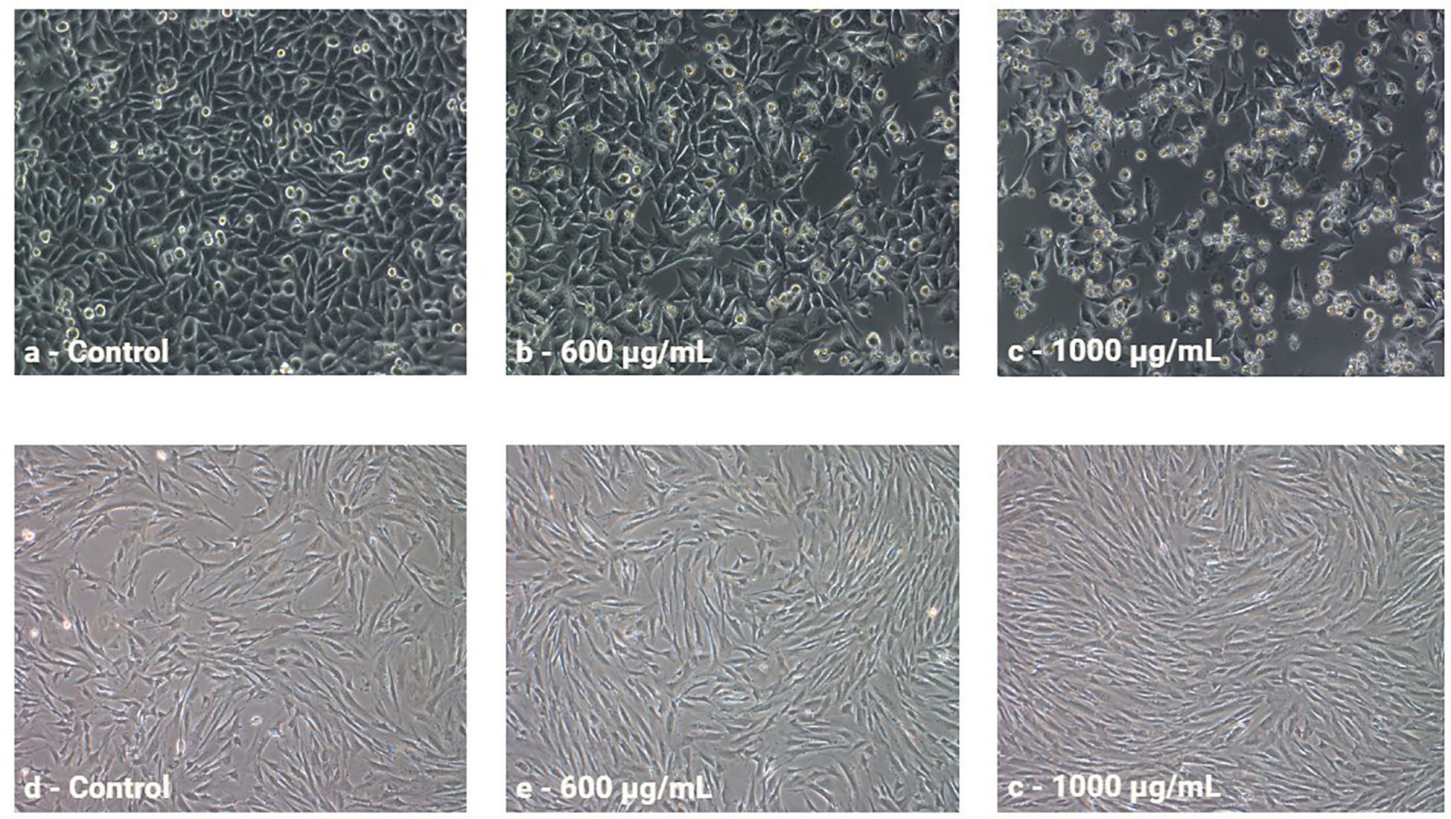
Morphology of A375 cells non-treated **(A)** and treated with the extract from Lili Marleen cv. **(B, C)** and Hs27 cells non-treated **(D)** and treated with the extract from Lili Marleen cv. **(E, F)**.

To evaluate cytotoxic activity, the melanoma cell line A375 and the normal skin cell line Hs27 were treated with increasing concentrations of anthocyanin-rich fractions derived from roses. Cell viability was subsequently assessed using the MTT assay. All values were expressed as a percentage of the control sample. The antiproliferative effect of rose extracts on the tumor cell line was evaluated positively due to the inhibition on cell growth. This effect was quantified by calculating the difference between the proliferation value of the control culture and the proliferation value of the extract-treated culture. At the same time, the effect on the normal cell line was interpreted with positive values, suggesting the ability of the extracts to stimulate the growth and regeneration of normal cells. This effect was quantified by calculating the difference between the proliferation value of extract-treated culture and the proliferation value of the control culture. Graphical illustrations of cell proliferation were used as means to more clearly demonstrate the cell evolution following treatment administration. For Porta Nigra variety, only positive values were recorded on the A375 tumor cell line, with stronger antiproliferative effect as concentrations increased (**Figure 8**). On the normal Hs27 cell line, all concentrations exhibited a proportional increase in proliferative effect with rising concentrations. Similarly, for Schwarze Madonna cultivar, the obtained values show positive and concentration-dependent increase of activity on the A375 cell line, which was slightly higher at each concentration compared to Porta Nigra cultivar (**Figure 8**). Nevertheless, there was no linear increase, the activity values remained similar up to 400 µg/mL, increased significantly at 600 µg/mL, followed by non-significant differences at subsequent concentrations. Conversely, on the Hs27 cell line, a positive impact is demonstrated. However, for Schwarze Madonna variety, concentrations of 800-1000 µg/mL indicate a slight decrease in proliferation, compared to the Porta Nigra variety. Furthermore, Lili Marleen cultivar recorded the highest values for the anti-proliferative effect on the A375 line (35.30 ± 1.76%) and the proliferative effect on the Hs27 line (46.72 ± 2.40%). Both tumor and normal cell lines demonstrated visible changes in cell morphology (**Figure 9**). Their characteristics noticeably changed after a 24-hour exposure to the treatment, becoming more visible as the concentration increased. The highest concentration of Lili Marleen rose extract led to significant cytotoxic effects in A375 tumor cell line, such as cell detachment, rounding up and shrinkage. On the other hand, Hs27 normal cells formed a confluent monolayer, exhibiting their typical elongated shape, without any sign of morphological stress markers.

### 3.4 Variability insights: principal component analysis

**Figure 10:**
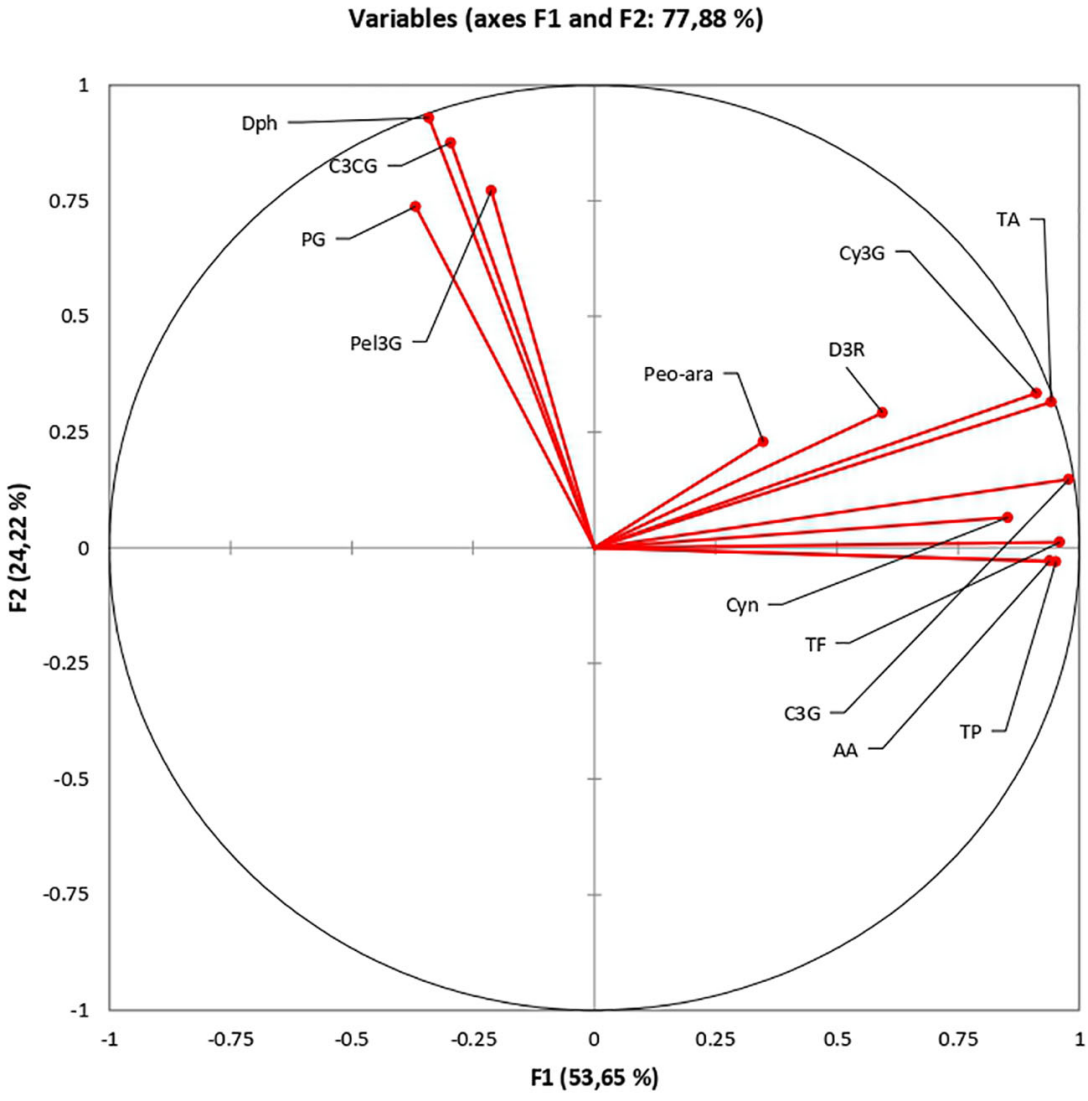
Exploring the biochemical relationships of roses: Principal Component Analysis.

Principal component analysis (PCA) provides mechanisms to describe relationships between different compounds. The results of the principal component analysis of rose biochemicals indicate a clear separation of cyanidin-coumaroyl-glucoside (C3CG), pelargonidin-glucoside (Pel3G), pelargonidin (Pg), and delphinidin (Dph) from the other compounds analyzed (**Figure 10**). The relationship between eigenvalues and principal components is presented in **Supplementary File**. This pattern could be attributed to shared structural features and biosynthetic pathways, distinct functional roles, unique metabolic regulation, or even analytical detection limitations. Further investigation of these compounds, including structural analysis, functional studies, and metabolic assessments, can help clarify the reasons for their varying concentrations. The scientific community can better understand their roles in rose coloration, potential health benefits, and mechanisms of resistance by exploring the biochemical relationships among these compounds.

### 3.5 Hierarchical clustering: dendrogram of rose varieties

**Figure 11:**
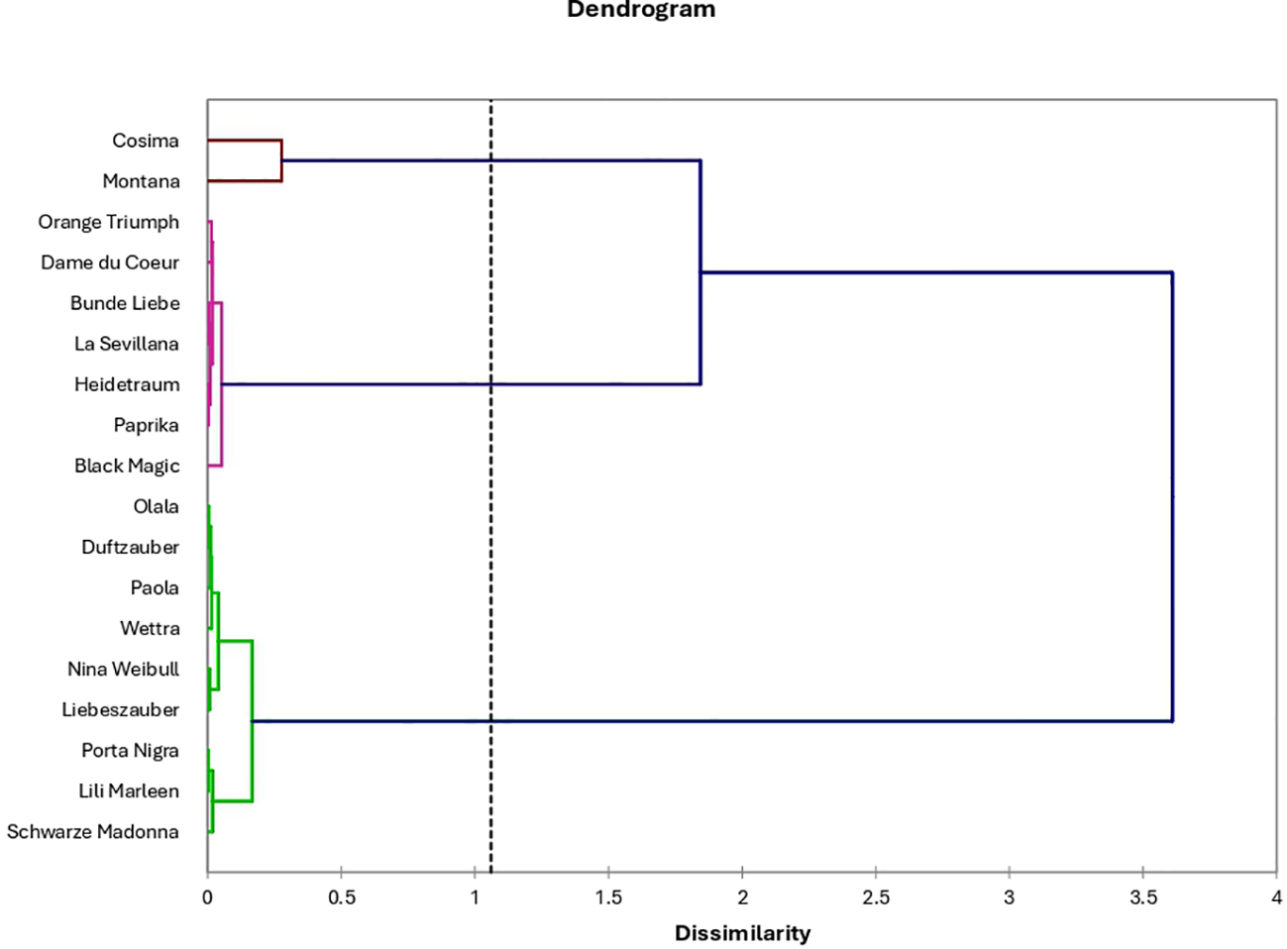
Hierarchical clustering of rose varieties based on total anthocyanin, total polyphenol, total flavonoid content, and individual anthocyanins concentration.

The hierarchical clustering of anthocyanins and phenolic compounds in roses provides critical insights into the complex relationships between these bioactive molecules, highlighting their structural similarities, biosynthetic pathways, and functional roles (**Figure 11**). By analyzing these clusters, we can identify patterns that suggest shared origins or functional linkages between different compounds, which are important for understanding how these molecules contribute to the overall chemical and biological properties of roses.

Cluster 1, which includes peonidin-arabinoside and delphinidin-rutinoside, suggests a close structural relationship between these two anthocyanins. This clustering indicates that these compounds may share a similar biosynthetic pathway, as structural similarities often correspond to shared enzymatic processes during their synthesis. Such relationships could reflect common metabolic precursors or enzymes involved in their formation, pointing toward a coordinated production mechanism in the biosynthetic network of anthocyanins in roses. Cluster 2 highlights a potential synergy between anthocyanins such as cyanidin-caffeoyl-glucoside, cyanidin-glucoside, and cyanidin, along with overall antioxidant activity and total phenolic content. This association suggests that these anthocyanins might work together with other phenolic compounds to enhance the antioxidant capacity of roses. Anthocyanins are known for their powerful antioxidant properties, and their interaction with other phenolics could amplify this effect, contributing to the health benefits and potential commercial value of rose-derived products. The functional role of these compounds in combating oxidative stress in plants may also reflect a broader ecological adaptation, where phenolics help protect against environmental stressors such as UV radiation or pathogen attack. Cluster 3 showcases the biochemical distinctiveness of several compounds, including delphinidin, cyanidin-coumaroyl-glucoside, pelargonidin-glucoside, and pelargonidin. The separation of these molecules into a distinct cluster suggests unique structural or functional properties that differentiate them from other anthocyanins in roses. This distinctiveness could be related to their individual roles in pigmentation, where each compound may contribute to different shades of color in rose petals, or their differential antioxidant or anti-inflammatory activities, which are influenced by slight variations in their molecular structure.

A greater understanding of these molecular relationships can enhance breeding programs aimed at developing rose cultivars with improved visual appeal and functional qualities. By selecting specific anthocyanin profiles, breeders could create roses with more vibrant colors, better resistance to oxidative stress, and greater health benefits in rose-based products. Therefore, the hierarchical clustering of anthocyanins and phenolic compounds acts as a valuable tool for guiding future research and practical applications in rose cultivation and product development.

### 3.6 Correlation assessment: heatmap visualization

**Figure 12:**
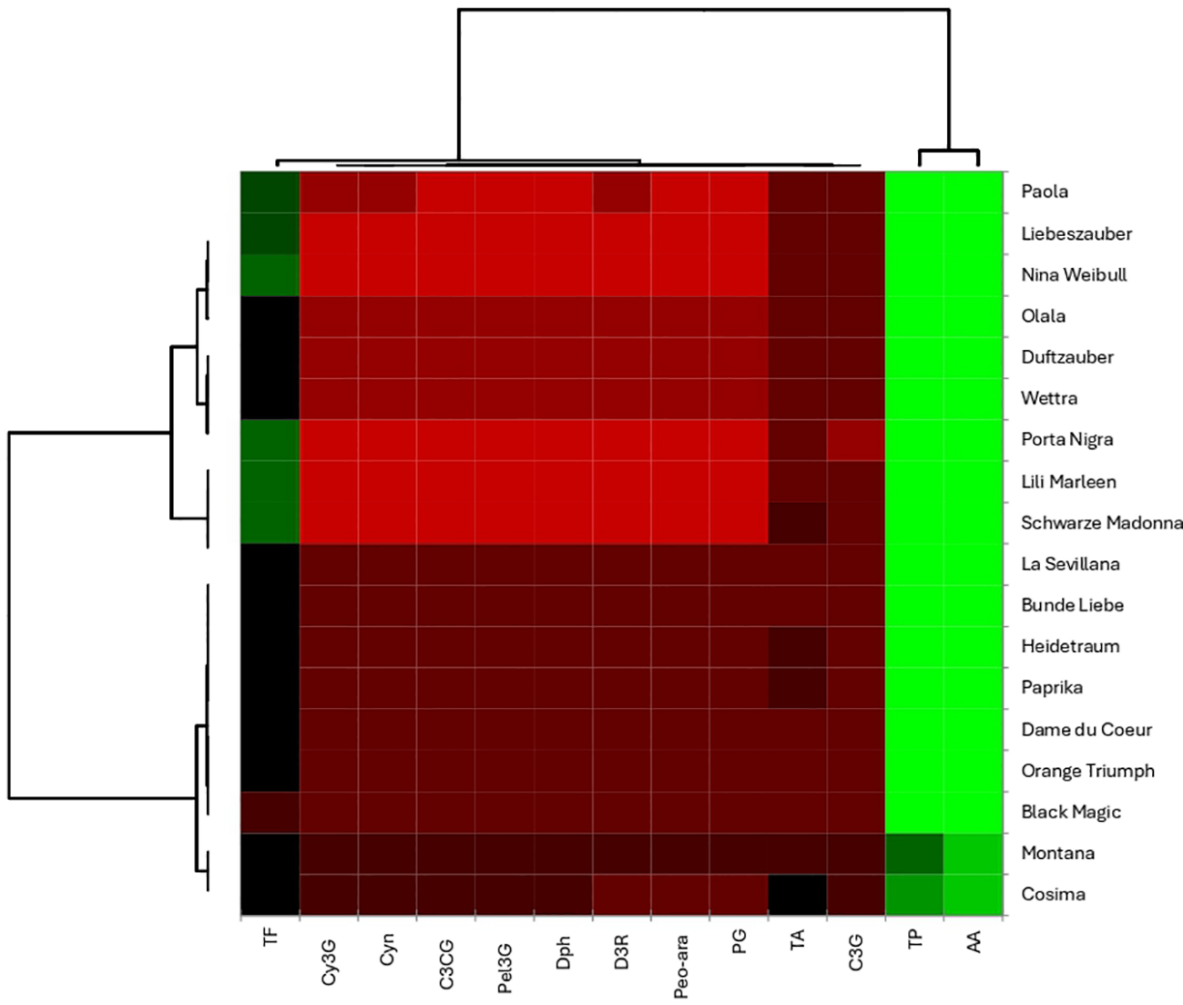
Heat map reveal distinct groups of roses with similar biochemical profiles.

The heatmap analysis in the current study provides a visual representation of the biochemical data from the rose varieties, allowing for the examination of relationships among various compounds. The results regarding the correlation assessment are presented in **Figure 12**. A key finding from the heatmap analysis of the rose biochemical data shows that total polyphenols (TP) and antioxidant activity (AA) tend to cluster together, which might suggest a connection between these two factors. This clustering also highlights a clear distinction among the different rose varieties. Specifically, the Paola, Liebeszauber, Nina Weilbull, Olala, Duftzauber, Wettra, Porta Nigra, and Lili Marleen varieties form a distinct group compared to Schwarze Madonna, La Sevillana, Brene de Liebe, Heidetraum, Paprika, Dame du Coeur, Orange Triumph, Blake Magic, Montana, and Cosima. This pattern indicates a potential link between the total polyphenol content and overall antioxidant activity in these rose varieties.

## 4 Discussion

Oxidative stress, generated by the accumulation of reactive oxygen species in the body, appears to be implicated in several pathological processes, including cancer, cardiovascular disease, and neurological disorders (Yoshihara et al., 2010). While numerous studies have demonstrated potential preventive effects of natural products against chronic diseases, bioactivities of many natural compounds are still unknown. Thus, a strong scientific basis for the effective application of natural compounds would come from comprehending and verifying their bioactivity as well as the underlying molecular mechanisms. In this study, a biochemical examination of extracts derived from rose cultivars was conducted, with a detailed analysis of antiproliferative activity performed on three selected extracts. The results of this study contribute important insights to the existing research by highlighting the potential health benefits of the rose varieties studied in this research. By identifying specific cultivars with strong bioactive properties, we create opportunities for further investigation into the therapeutic uses of roses. This research emphasizes the need for ongoing studies into the antioxidant and antiproliferative effects of plant-based compounds, which may lead to new, natural approaches for preventing and treating diseases related to oxidative stress. The HPLC analysis of the rose petal samples identified a total of 9 anthocyanin compounds. The most common and abundant anthocyanin found in the rose cultivars evaluated in this study was cyanidin-caffeoyl-glucoside. Moreover, cyanidin-coumaroyl-glucoside, cyanidin-glucoside, and pelargonidin-glucoside were also among the major anthocyanins in some varieties. This may be explained by the stability of the anthocyanins, which has been shown to be dependent on the type of sugar that is attached to an aglycone, while glucosides might be more resilient to mechanisms that break down anthocyanins, such as hydrolysis (Ichiyanagi et al., 2001). As there are differences between rose cultivars, their anthocyanin composition is also highly variable. As an example, Mikanagi et al. (2000) discovered 12 anthocyanin compounds in 52 rose varieties, while only two common anthocyanin compounds (cyanidin-glucoside and pelargonidin-glucoside) were detected in the rose cultivars evaluated in this study. The percentage of anthocyanins with attached glucosides was higher than those which had different types of sugar, such as rutinoside or sophoroside. In the *Rosa hybrida* cv. Noblered variety, only two anthocyanins (cyanidin-di-glucoside and pelargonidin-di-glucoside) were found (Lee et al., 2011). The amount of total anthocyanins in our cultivars ranged from 12.42 to 331.95 mg CGE/100g FW. Similar results were found in a study of 9 rose cultivars, anthocyanin concentrations ranging from 0.61 to 502.64 mg CGE/100g FW (Yang and Shin, 2017). The study also concluded that the characteristic flower color of every cultivar was responsible for these variations between the cultivars. In another study, anthocyanin content varied from 164 to 180 mg CGE/100g FW based on the year the roses were picked (2011–2013) (Cendrowski et al., 2017). Moreover, in a study conducted by Cunja et al. (2014) on 6 rose cultivars and species, total anthocyanin content ranged between 1.39 and 85.58 mg/100g FW. Furthermore, there might be a strong correlation between the color intensity and the accumulation of anthocyanins and other polyphenols in rose (*Rosa x hybrida*) petals (Schmitzer et al., 2009). For instance, cyanidin-3,5-di-O-glucoside was found to be responsible for the red coloration and their lightness in coloration in rose petals. Additionally, the concentration of this anthocyanin decreased with 26.07% in senescent flowers compared to bud stage. Moreover, Schmitzer et al. (2010) analyzed the biochemical composition of 8 groundcover roses during flower development. The anthocyanin content was low during the bud stage, nearly doubled when the flower partially opened, and reached the highest content when the flower fully opened. Besides, senescence was associated with a slight decrease in total anthocyanin content. In another study, among 6 rose varieties, the concentration of anthocyanins in petals increased initially, and then decreased during the stages of floral development (Wan et al., 2019). The growth of petal cells and degradation of anthocyanins may be responsible for the decreased concentration of anthocyanins (Luo et al., 2017). Furthermore, in 50 rose varieties from India, collected in different growing seasons (December and March), lower anthocyanin content was observed for all the varieties in March as compared to December (Kumari et al., 2017). The analysis of each developmental stage of these rose cultivars was not included in current study, but it remains an intriguing perspective for further investigation.

Anthocyanins have been linked to a variety of stress response pathways in plants (Kaur et al., 2023). A high antioxidant activity is an indicator of the plant’s high tolerance to stress, triggered by environmental factors such as high light intensity, drought, and extreme temperatures. Furthermore, improved resistance to oxidative stress through increased anthocyanin concentration may result in salt tolerance (Eryılmaz, 2006) and drought tolerance (Hinojosa-Gómez et al., 2020). Since anthocyanin molecules are diverse and stress response pathways are complex, it is difficult to identify which particular compounds are primarily responsible for stress tolerance. However, some studies suggest that certain compounds may be involved in stress tolerance. A defense mechanism against oxidative damage to cellular structures was demonstrated by cyanidin-3-glucoside (Daiponmak et al., 2010). Moreover, the type of sugar residues of anthocyanidins is important for physiological functions (Rubinskiene et al., 2005; Reen et al., 2006). According to Eguchi and Sato, 2009, in the higher nodal position, which is exposed to extreme UV stress, tartary buckwheat may produce cyanidin-3-rutinoside at a high ratio, potentially increasing its antioxidant activity. Although cyanidin-3-rutinoside was not detected in our rose cultivars, some of them might have an increased tolerance to abiotic stress factors since they have a significant amount of cyanidin-3-glucoside. However, in addition to the limited data regarding the type of anthocyanin compound responsible for tolerance to various stress factors, the literature does not contain any studies examining the correlation between anthocyanin concentration and their ability to withstand abiotic stress in roses. Consequently, the results of the current study may open new research opportunities and simultaneously stimulate interest within the scientific community in the near future.

The total polyphenolic content in the current study ranged between 208 and 2600 mg GAE/100g FW, total flavonoid content between 102 and 705 mg CE/100g FW, whereas the antioxidant activity values ranged between 304 and 1403 µmol TE/g FW. The results on the biochemical composition of the rose cultivars studied are consistent with reports from previous studies on different rose varieties. Yang and Shin (2017), in their study on 9 edible rose varieties cultivated in Jincheon (South Korea), reported the total polyphenol content to be between 798.67 and 2978.89 mg GAE/100g FW and total flavonoid content was between 78.64 and 531.54 mg CE/100g FW. Moreover, in a study conducted by Ge and Ma (2013), the rose variety from Yunnan province (China) identified a total polyphenol content of 2087.43 ± 17.37 mg GAE/100g FW. In another study that analyzed 10 rose varieties, the total polyphenols contents ranged from 350 ± 81 to 1196 ± 28 mg GAE/100g FW, whereas total flavonoid contents ranged from 25.7 ± 1.6 to 120 ± 11 mg CE/100g FW (dos Santos et al., 2018). Furthermore, Trinh et al. (2018) conducted a study on rose extract and revealed antioxidant activity values ranging from 873.4 to 1635.6 µmol TE/g FW. Additionally, Rop et al. (2012) reported a total phenolic content of 502 ± 34 mg GAE/100g FW on a tea rose variety (*Rosa odorata*). However, there are some rose species with significantly higher content of polyphenols than our rose varieties. Kumar et al. (2009) reported values for total phenolic content ranging between 14500 and 25400 mg GAE/100g FW in three rose species (*R. damascena, R. bourboniana, R. brunonii*).

Despite extensive research conducted on the antiproliferative potential of anthocyanins, only a few studies have been performed on anthocyanins from roses (Netzel et al., 2007; Zu et al., 2010; Zamiri-Akhlaghi et al., 2011; Rivas-García et al., 2021). The study aimed to investigate the bioactivity of anthocyanin-rich extracts from roses and their potential cytotoxicity on normal human skin cells. The qualitative differences between cultivars are also reflected in their antiproliferative capacity on cell lines. Lili Marleen cv. demonstrated an especially positive impact on both tumor and normal cell lines thus far, while not being the variety with the highest concentration of total anthocyanins or total polyphenol content among the extracts selected for further investigation on cell lines. Moreover, Schwarze Madonna variety demonstrated a higher concentration of total anthocyanins compared to both Lili Marleen and Porta Nigra, as revealed by the statistical analysis. A comparison of Lili Marleen, Schwarze Madonna, and Porta Nigra varieties revealed significant differences in antiproliferative activity, despite no statistical differences in total polyphenols, total flavonoids, or antioxidant activity. When compared with two other rose cultivars such as Schwarze Madonna and Porta Nigra (also with high anthocyanin content), the Lili Marleen cultivar demonstrated significantly higher antiproliferative activity (concentration used as 1000 µg/mL), as well as enhanced proliferative activity at concentrations ranging from 400 to 1000 µg/mL. Polyphenol or anthocyanin complexes may explain their cytotoxic effect. Nowak et al. (2014) evaluated various solvents for rose extracts. They found that the water fraction, after the removal of most phenolic acids and flavonoids, exhibited cytotoxic activity on cancer cell lines without harming normal skin fibroblasts. Although they suggested that the activity of water fraction might be related to condensed tannins, polysaccharides, or different complexes of proteins, our study rules out this possibility since our extracts have been purified both on Amberlite XAD-7 and Sephadex LH-20 columns. Nevertheless, they did not analyze the anthocyanins profile in those extracts. Vishnu et al. (2019) showed that the presence of acylated anthocyanins played a vital role in antiproliferative activity of an extract from sweet potato on three cancer cell lines. However, Konczak-Islam et al. (2003) demonstrated that non-acylated anthocyanins from sweet potato exhibited anti-cancer activity in human promyelocytic leukemia HL-60 cells. Additionally, Jing et al. (2008) reported that anthocyanins with pelargonidin, triglycoside, and/or acylation with cinnamic acid were not as effective anticancer agents as non-acylated monoglycosylated anthocyanins. Cyanidin-coumaroyl-glucoside was only detected in Porta Nigra cv., and cyanidin-caffeoyl-glucoside had the highest concentration in Schwarze Madonna cv. Therefore, acylated anthocyanins could not have influenced the Lili Marleen extract in the current study. Anticancer activity is influenced by the type of aglycones, sugars, acylated acids, and the location and degree of glycosylation and acylation (Li et al., 2017). Rossetto et al. (2002) suggested that substances with relatively weak bioactivity could become more effective when used in combination. Therefore, a synergistic effect of some anthocyanins might be responsible for the underlying mechanism and improved impact of Lili Marleen cv. However, more research is needed to fully understand the structure–activity relationship of anthocyanins, as well as the synergistic effect of their interaction.

The study has some limitations. Rose extracts were analyzed only on skin cell lines. Since different cell lines and tissues possess distinct genetic compositions, receptors and signaling pathways, they may react differentially to the same anthocyanin-rich extract. Furthermore, this study only addresses *in vitro* antiproliferative activity. *In vivo* experiments are needed to further strengthen the potential *in vitro* results, though the low bioavailability of anthocyanins could complicate the conduct of such studies. Different factors such as pH, temperature, light, solvents, presence of oxygen, enzymes, other flavonoids, proteins, and metallic ions can also affect the stability of anthocyanins (Fang, 2014). However, anthocyanins could be applied locally and avoid gastrointestinal absorption with various pH fluctuations.

This comprehensive biochemical analysis of 18 rose (*Rosa* L.) cultivars has provided valuable insights into their diverse chemical compositions. The novelty of this study is highlighted by the few existing literature data regarding anthocyanins from rose petals and their biological activity tested on these cell lines is missing from the literature, as far as we know. Through HPLC-ESI-MS analysis, 9 individual anthocyanins were identified. The varieties identified with the highest concentrations of anthocyanins also showed the highest values for total polyphenols, total flavonoids, and antioxidant activity, without significant differences among these varieties. Principal component analysis (PCA) identified distinct groups of rose varieties with similar biochemical profiles. This analysis also pinpointed key compounds, like total polyphenols in some cases, that significantly influence the overall biochemical diversity of roses. The dendrogram’s hierarchical clustering indicates that all rose varieties likely share a common ancestor, followed by various diversification events. This helps explain the different clusters observed, where some varieties maintain a more ancestral biochemical profile (Cluster 3), while others (Clusters 1 and 2) have evolved differently due to genetic or environmental factors. The heatmap analysis revealed a potential link between total polyphenol content (TP) and antioxidant activity (AA). This suggests that varieties with higher total polyphenols may exhibit stronger antioxidant activity. Further research is needed to confirm this association and understand the specific types of polyphenols contributing to antioxidant potential. These conclusions, based on PCA, dendrogram analysis, and heatmaps, provide valuable insights into the biochemical relationships, diversity drivers, and potential health benefits of various rose varieties. The subsequent examination of three specific cultivars through cell culture studies demonstrated a dual impact by inhibiting A375 cancer skin cells while simultaneously promoting cell proliferation in Hs27 normal skin cells in a concentration-dependent manner. Among the three tested varieties, Lili Marleen cv. showed an outstanding effect, exhibiting the highest level of inhibition of A375 cell line and the most significant promotion of Hs27 normal cell proliferation. This dual functionality presents a promising avenue for further research into the potential medicinal applications of anthocyanins from rose cultivars.

## Data Availability

The original contributions presented in the study are included in the article/**Supplementary Material**. Further inquiries can be directed to the corresponding author.
