# Supplementary material for: Antiproliferative and biochemical evaluation of rose extracts: impact on tumor and normal skin cells
Source: Front Plant Sci. 2024 Oct 30;15:1477243. doi: 10.3389/fpls.2024.1477243 (PMC11557480; doi:10.3389/fpls.2024.1477243)
Supplement: Supplementary file 1 [file DataSheet1.pdf]

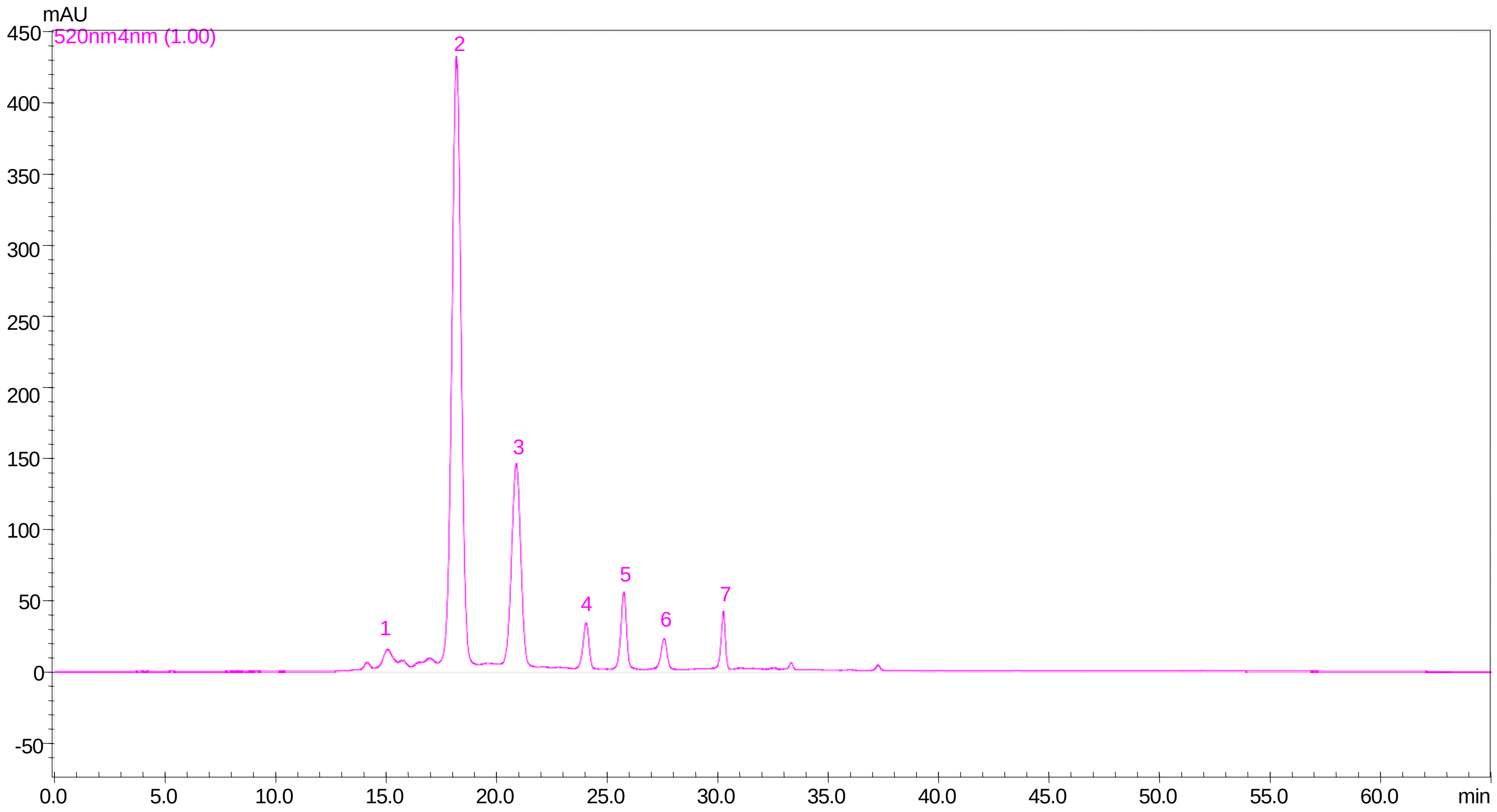


**Figure S1**. The HPLC Chromatogram for anthocyanin separation in **Paprika** cultivar

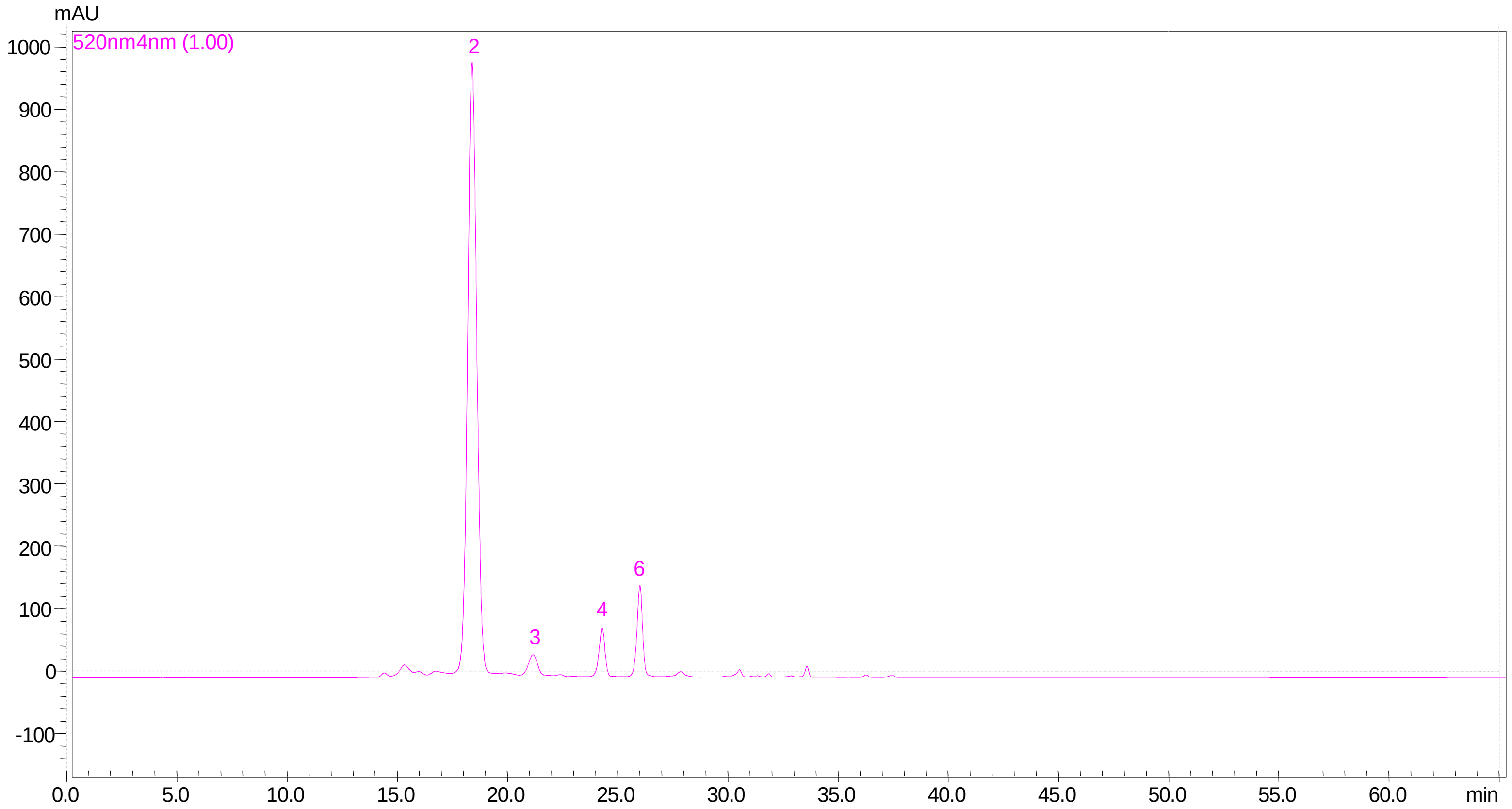


**Figure S2**. The HPLC Chromatogram for anthocyanin separation in **Duftzauber** cultivar

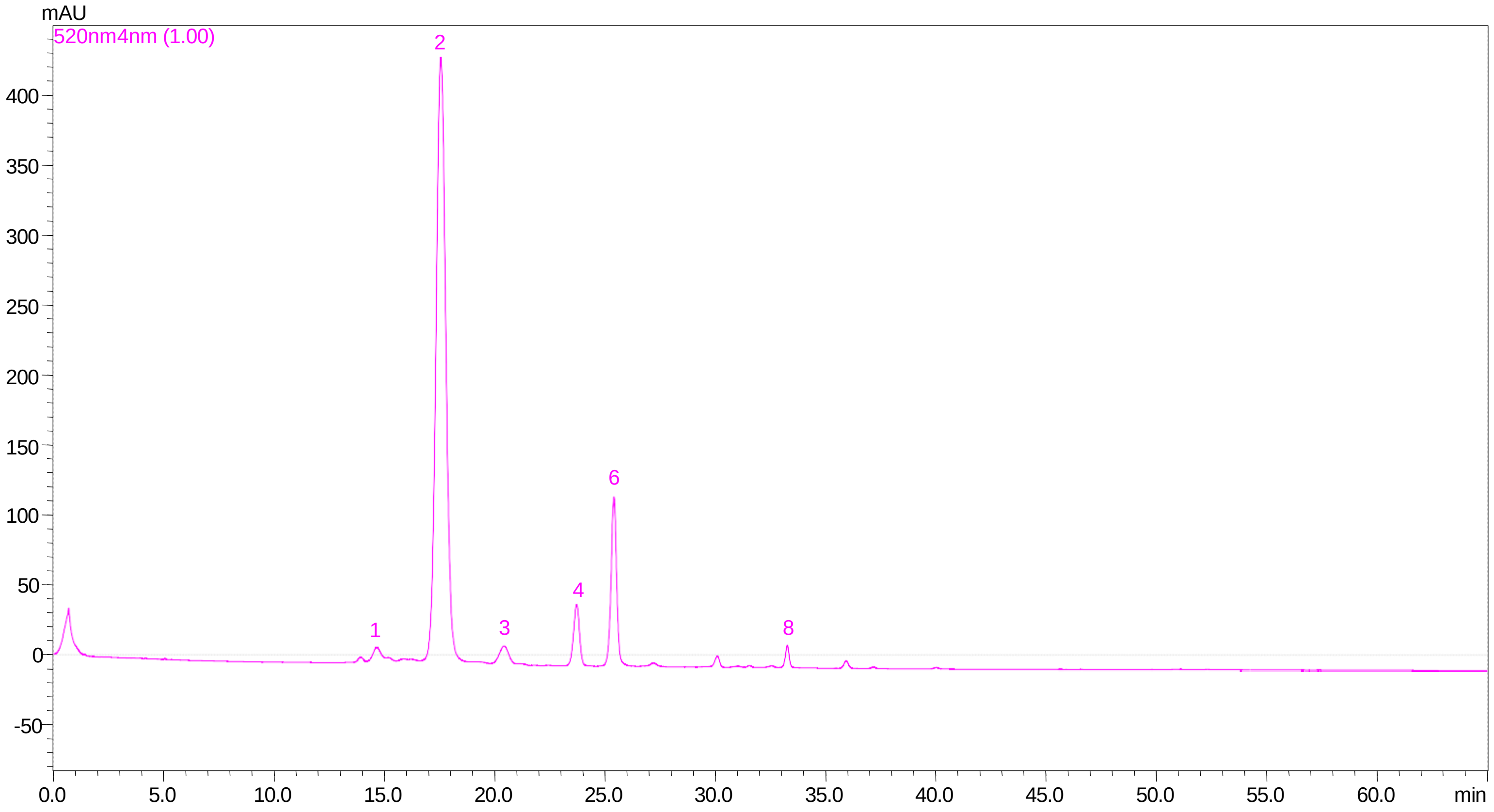


**Figure S3**. The HPLC Chromatogram for anthocyanin separation in **Heidetraum** cultivar

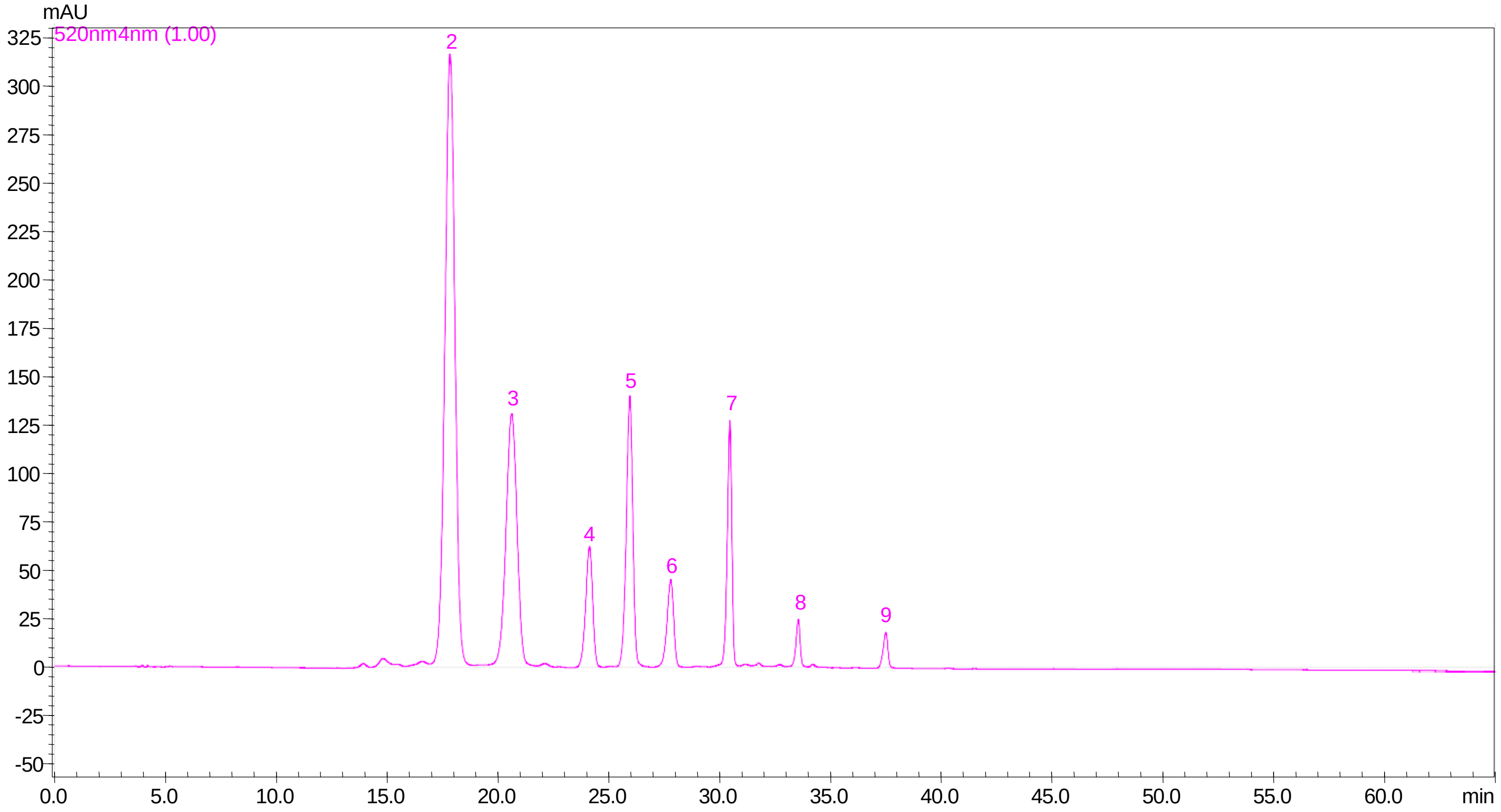


**Figure S4**. The HPLC Chromatogram for anthocyanin separation in **Montana** cultivar

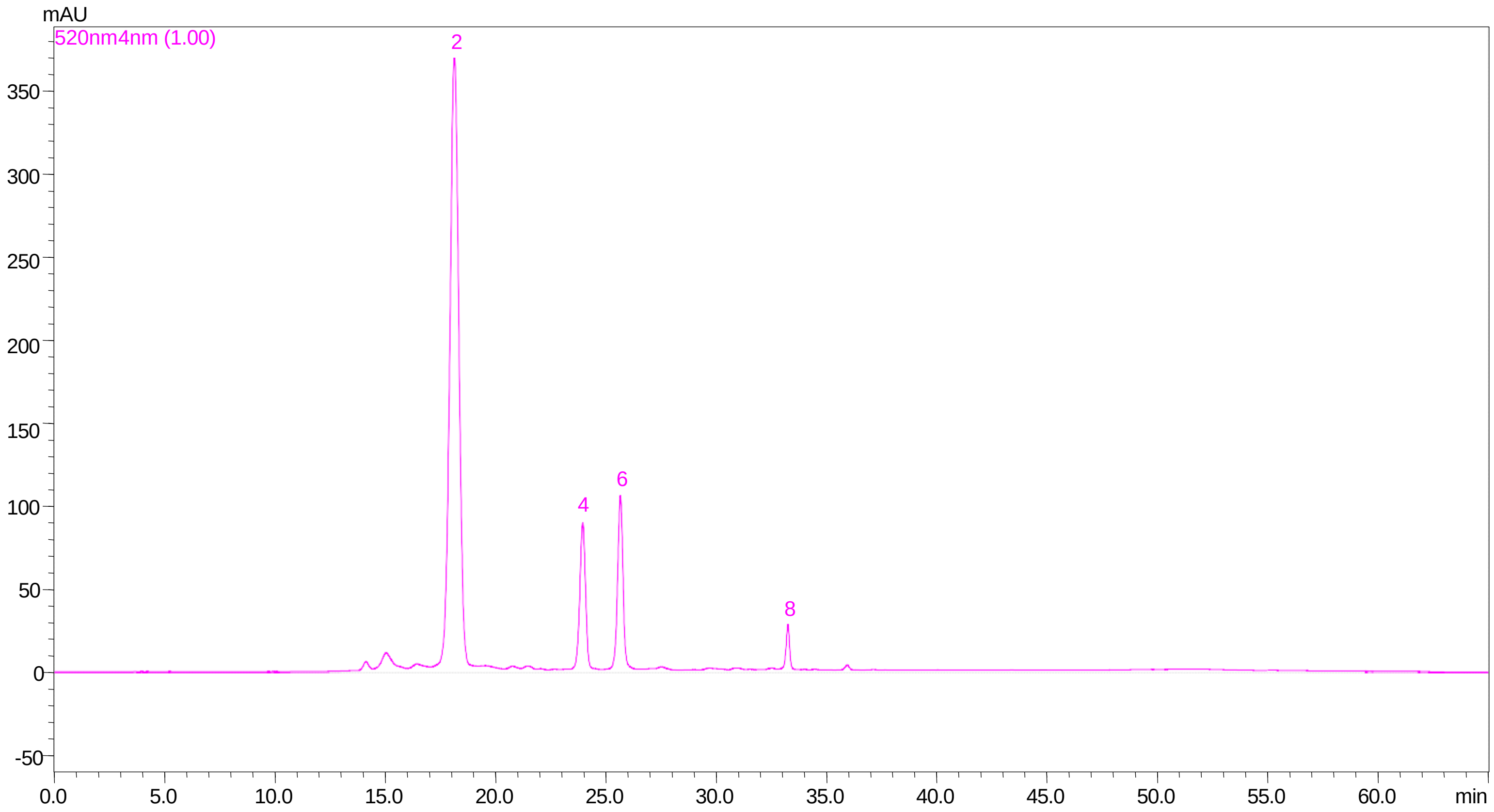


**Figure S5.** The HPLC Chromatogram for anthocyanin separation in **Dame du Coeur** cultivar

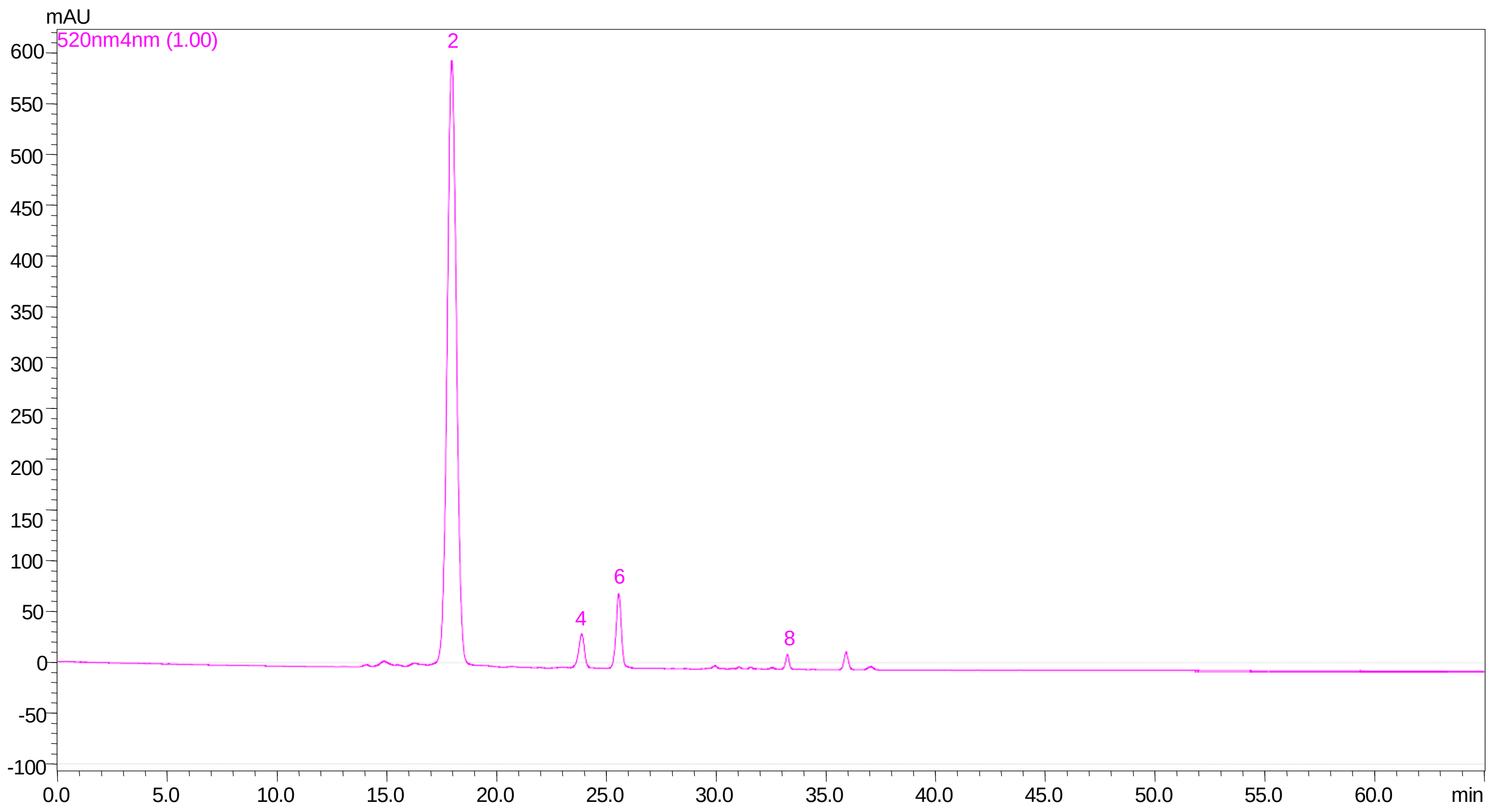


**Figure S6**. The HPLC Chromatogram for anthocyanin separation in **Wettra** cultivar

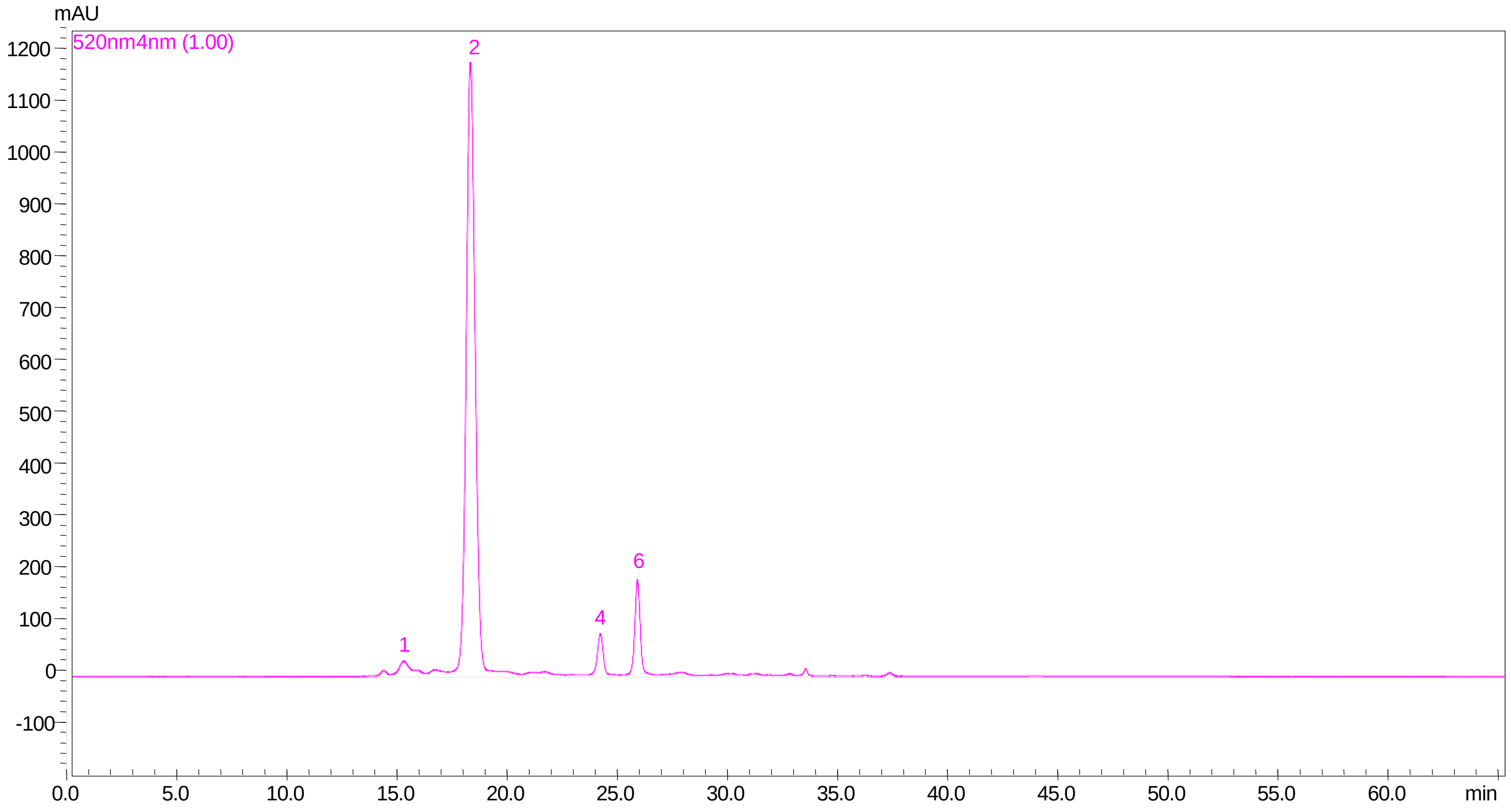


**Figure S7.** The HPLC Chromatogram for anthocyanin separation in **Paola** cultivar

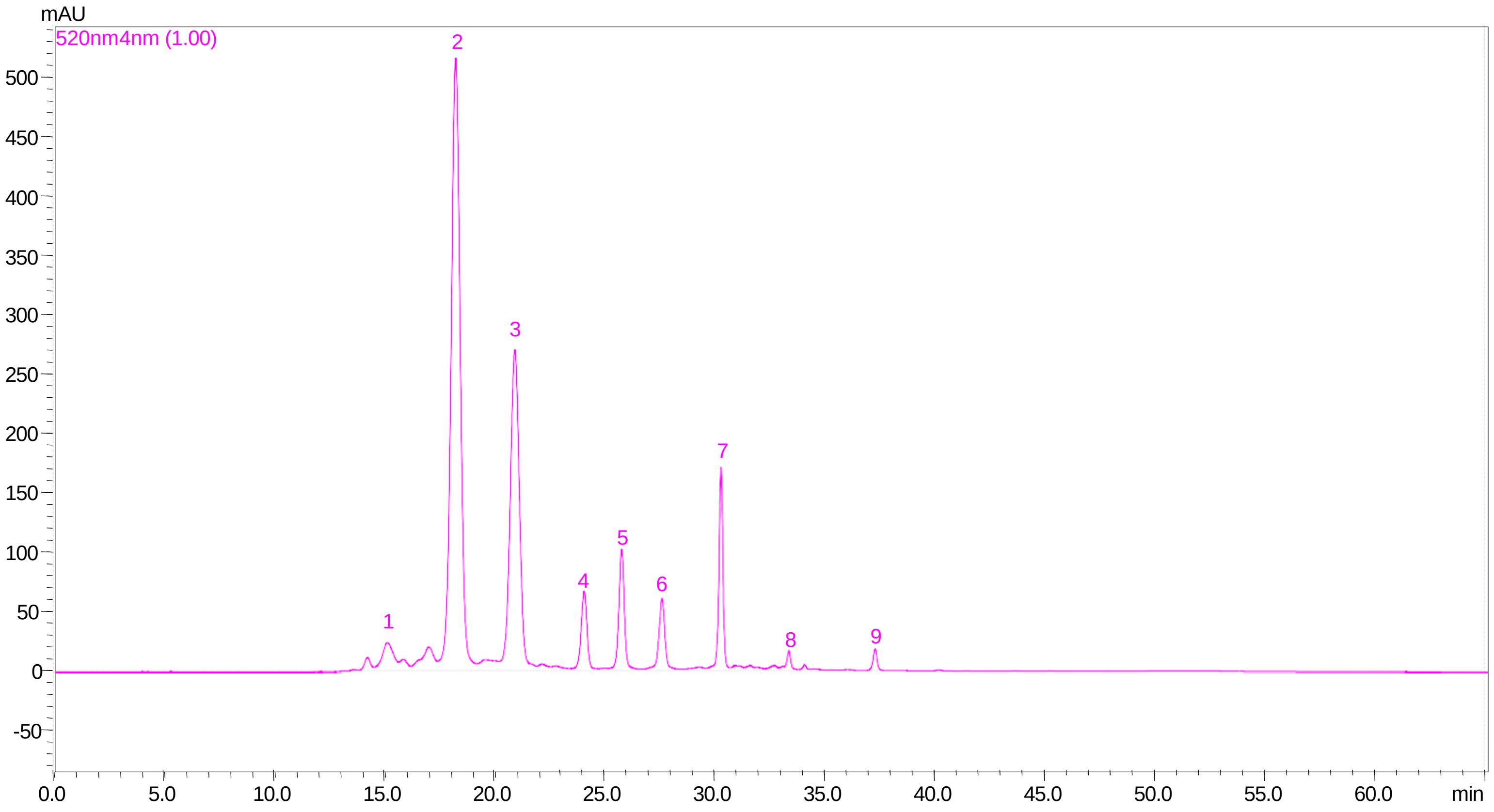


**Figure S8.** The HPLC Chromatogram for anthocyanin separation in **Cosima** cultivar

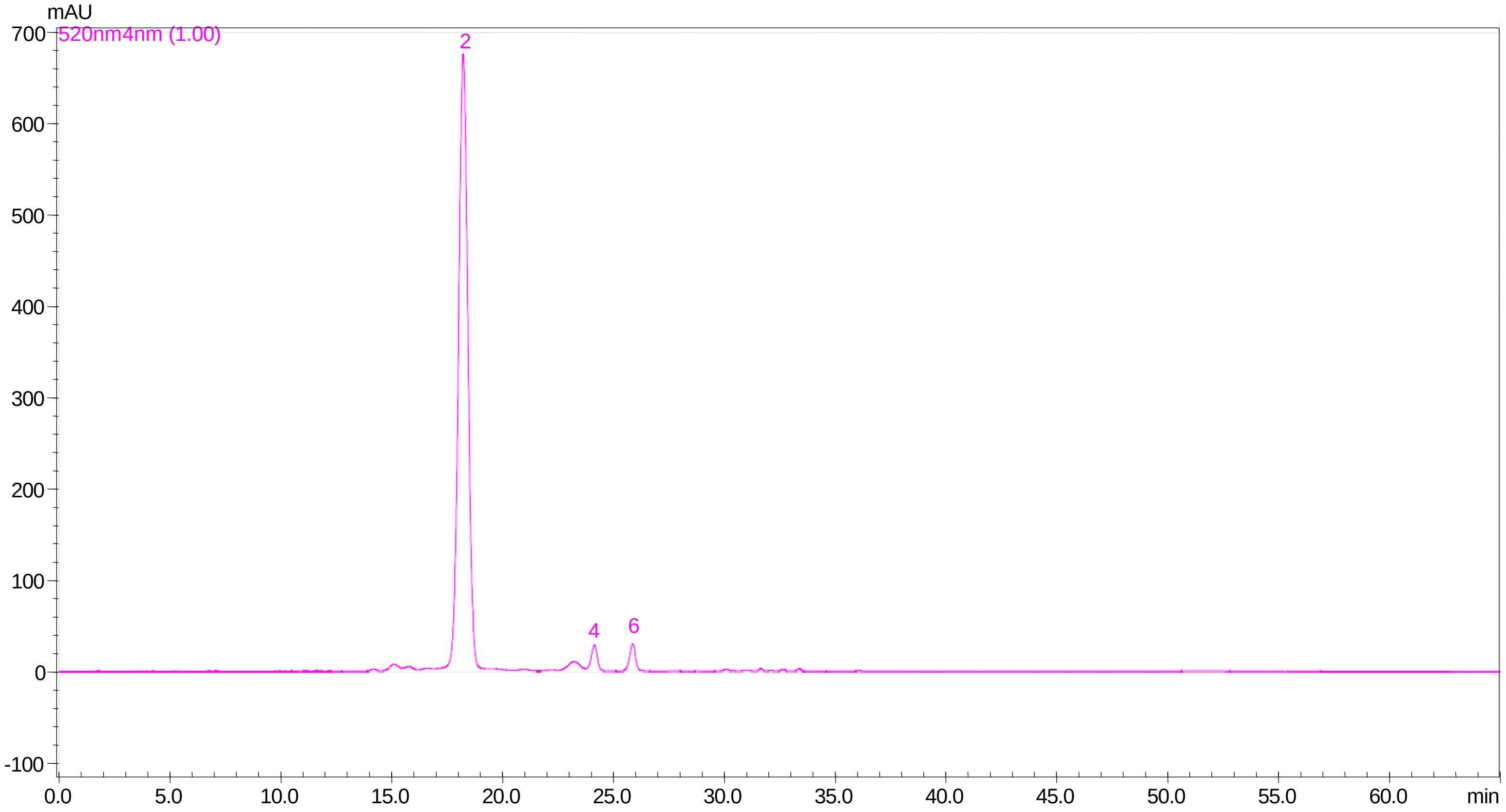


**Figure S9**. The HPLC Chromatogram for anthocyanin separation in **Olala** cultivar

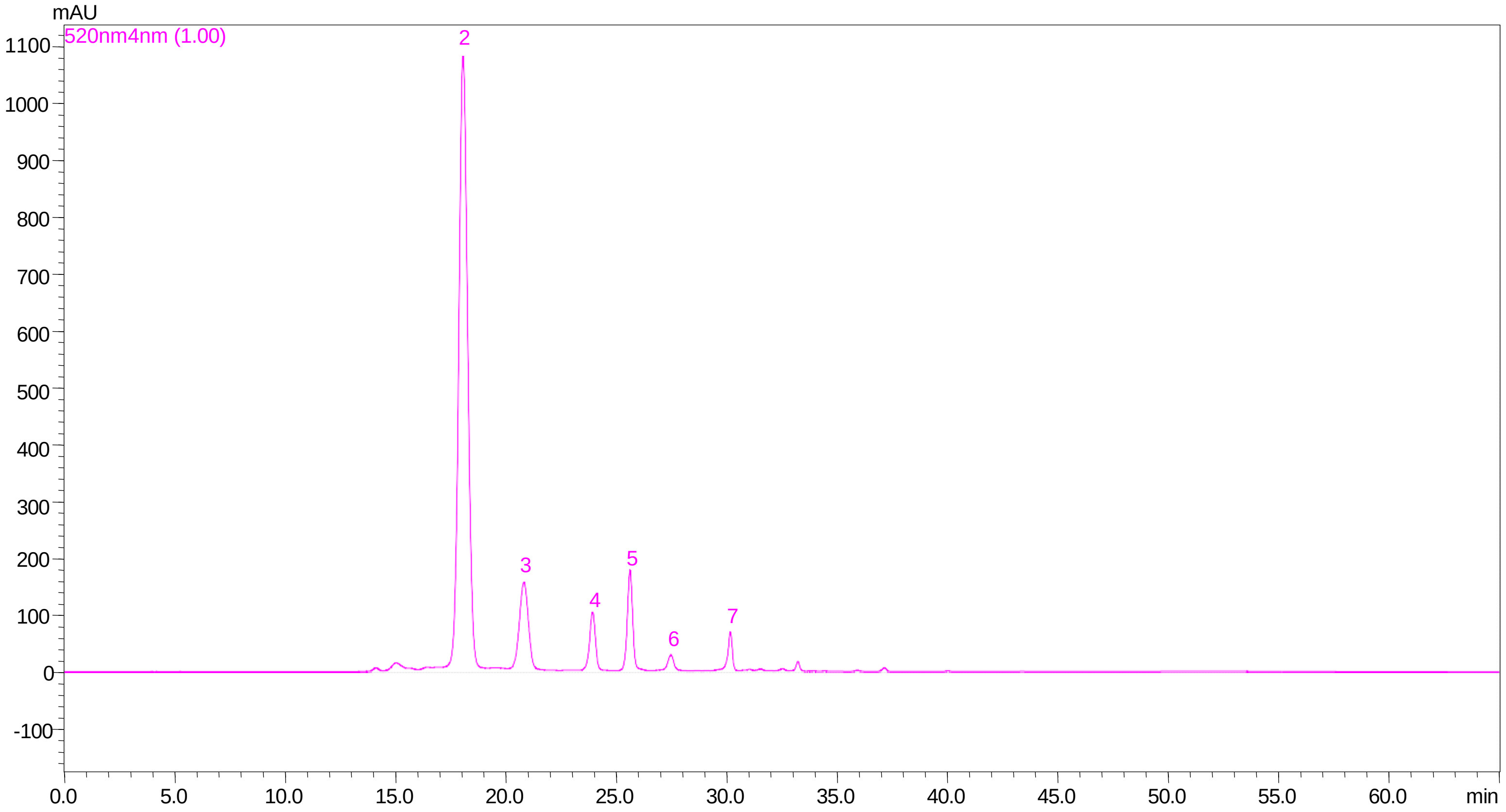


**Figure S10**. The HPLC Chromatogram for anthocyanin separation in **Libezauber** cultivar

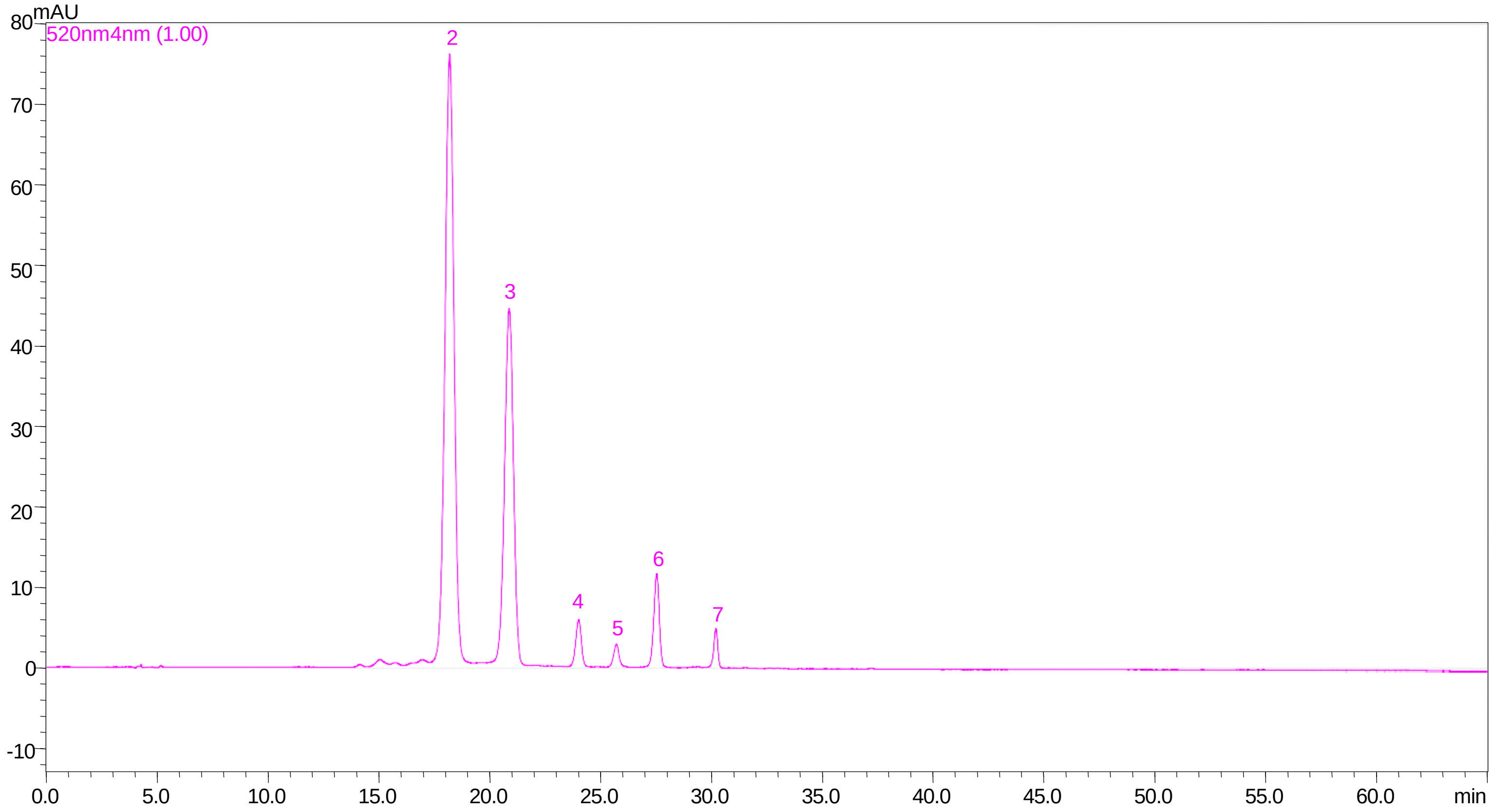


**Figure S11**. The HPLC Chromatogram for anthocyanin separation in **La Sevillana** cultivar

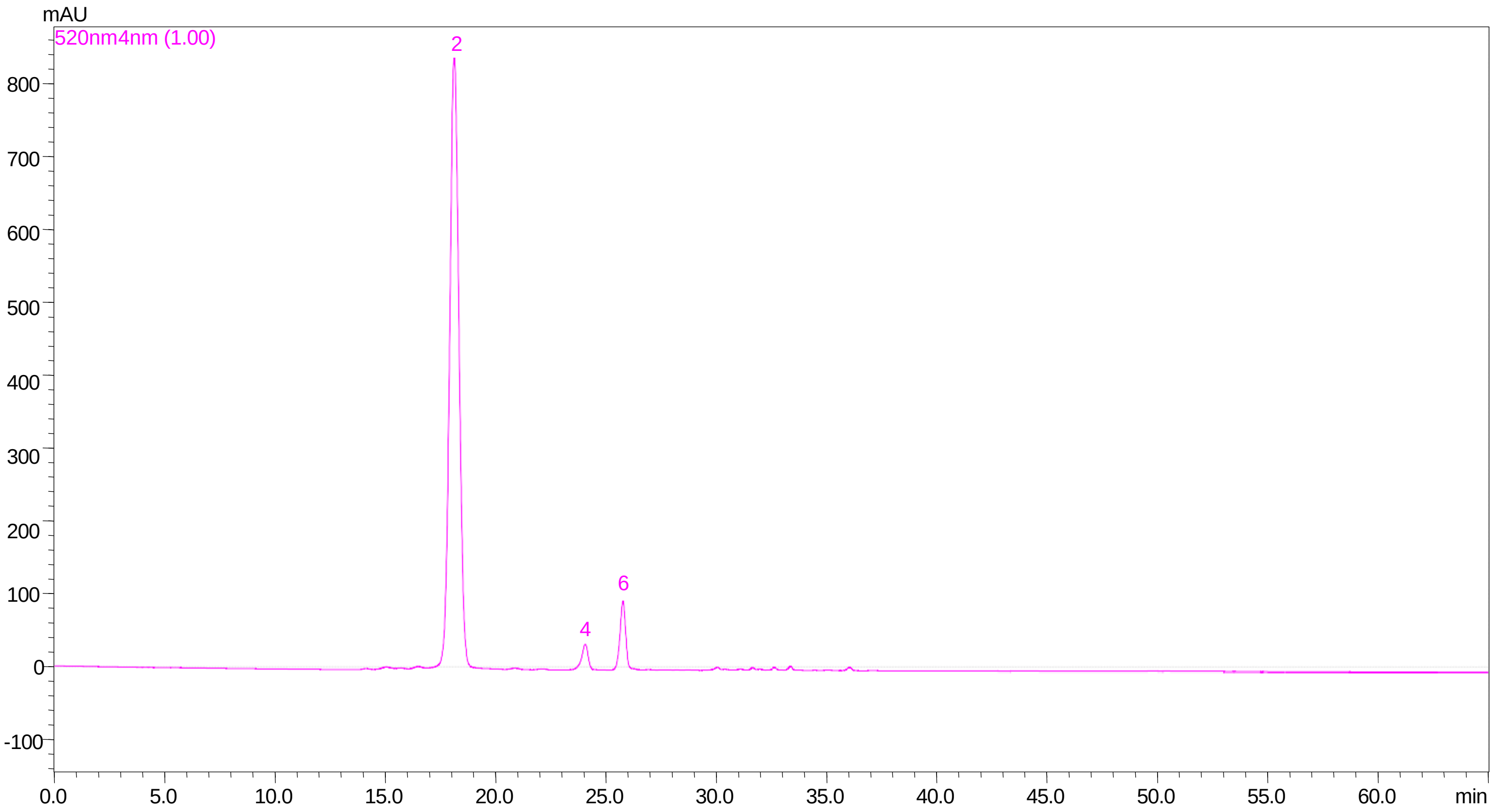


**Figure S12**. The HPLC Chromatogram for anthocyanin separation in **Brene de Liebe** cultivar

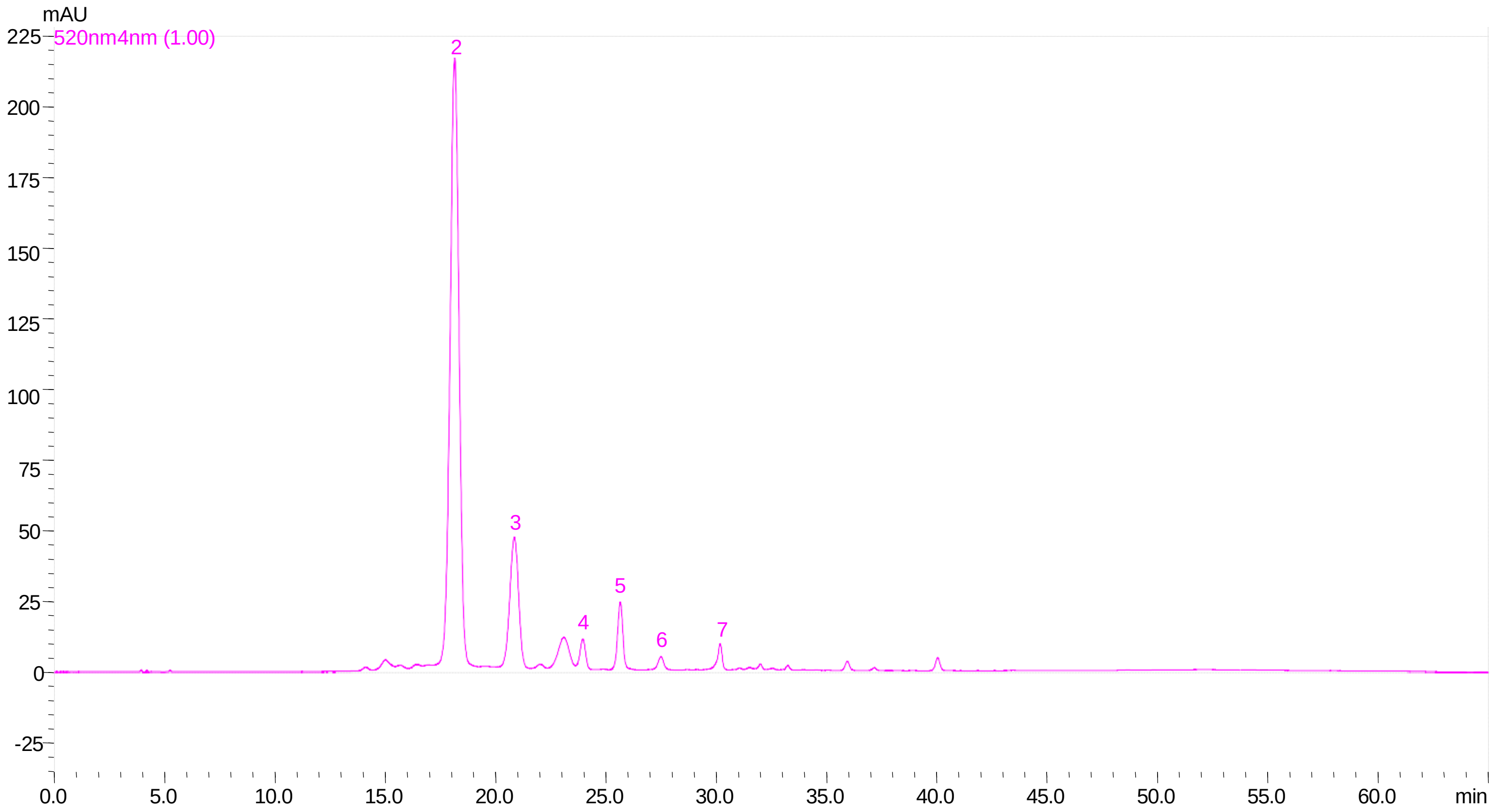


**Figure S13**. The HPLC Chromatogram for anthocyanin separation in **Orange Triumph** cultivar

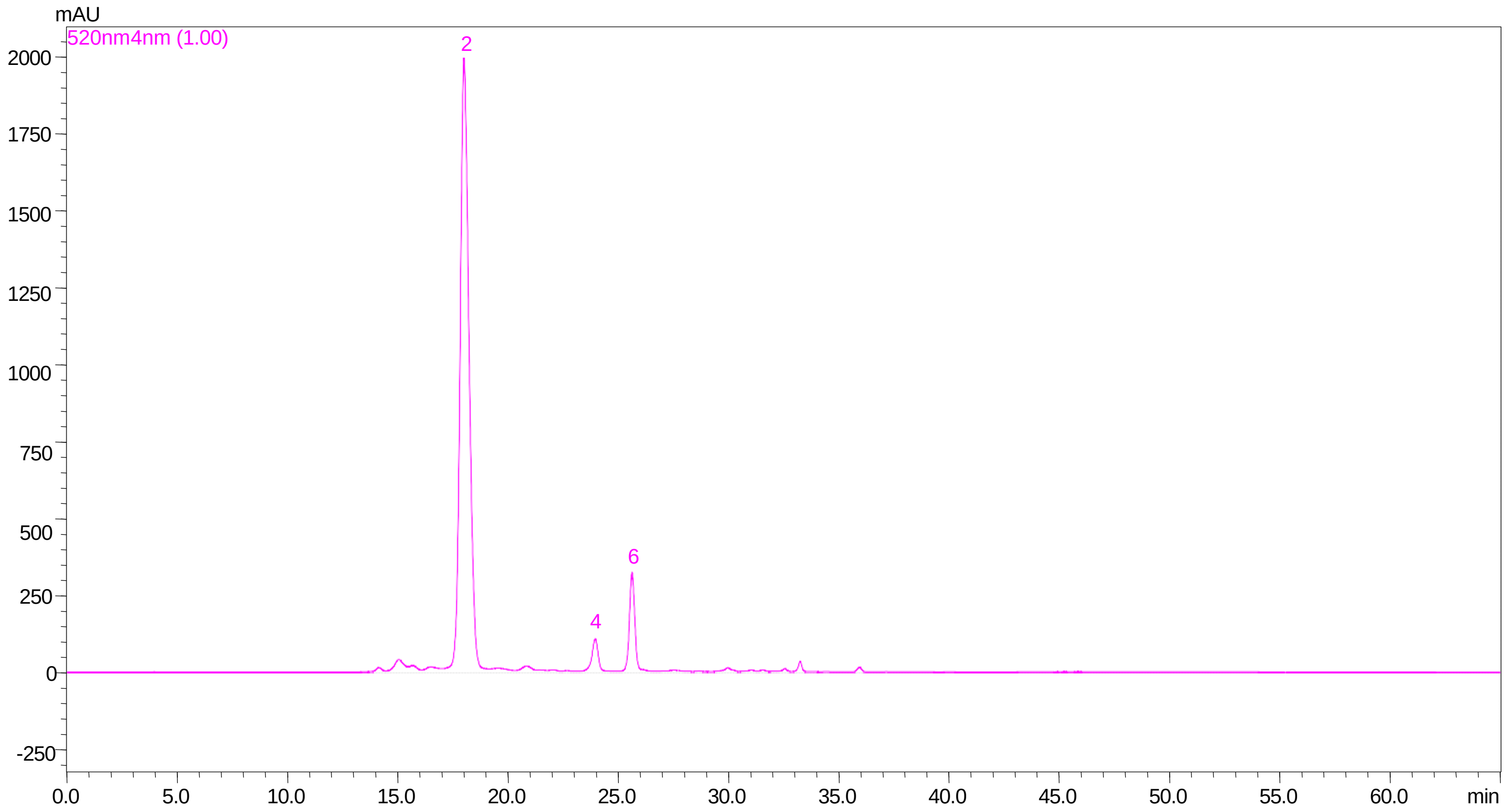


**Figure S14**. The HPLC Chromatogram for anthocyanin separation in **Lili Marleen** cultivar

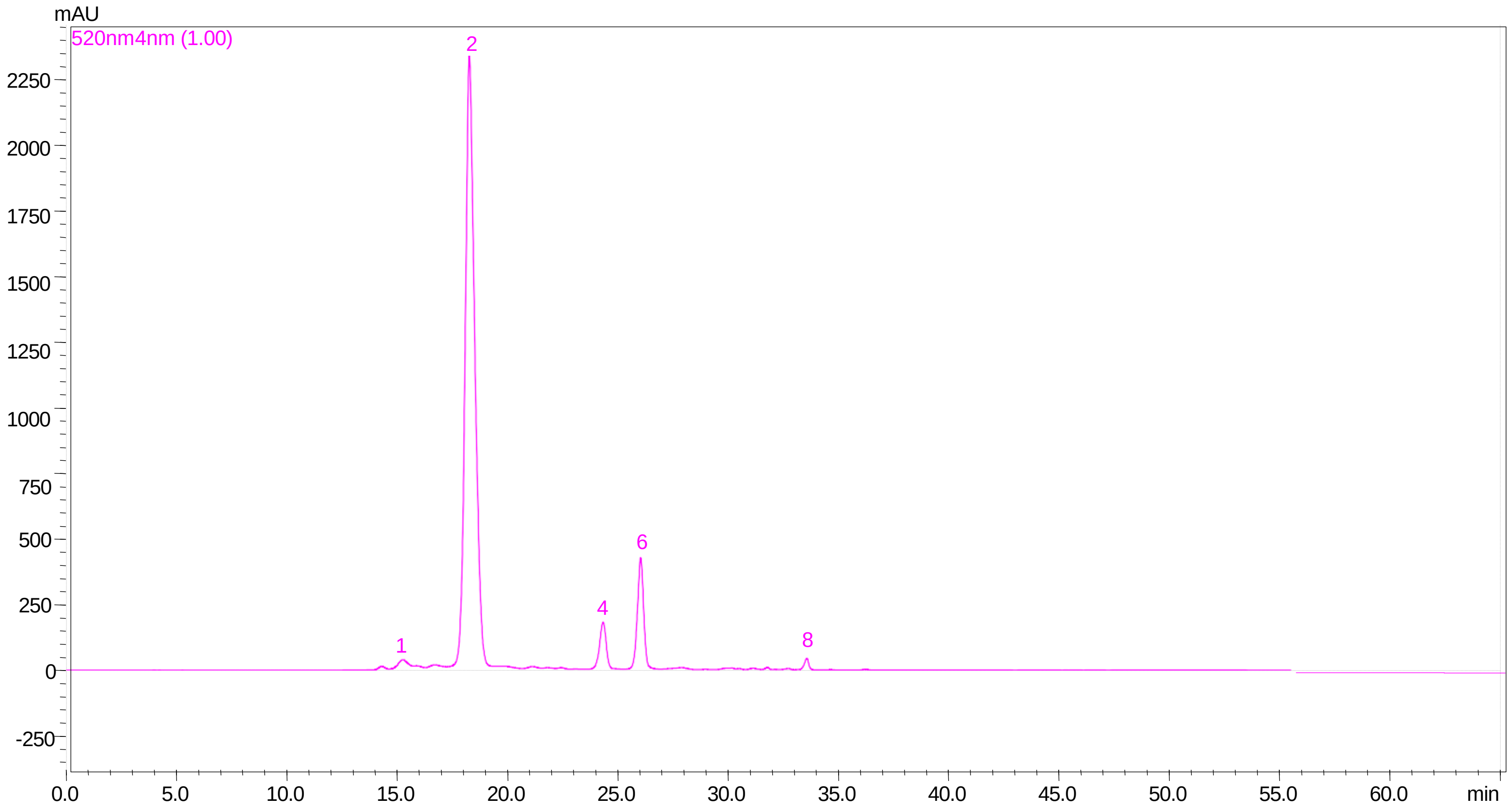


**Figure S15**. The HPLC Chromatogram for anthocyanin separation in **Schwarze Madonna** cultivar

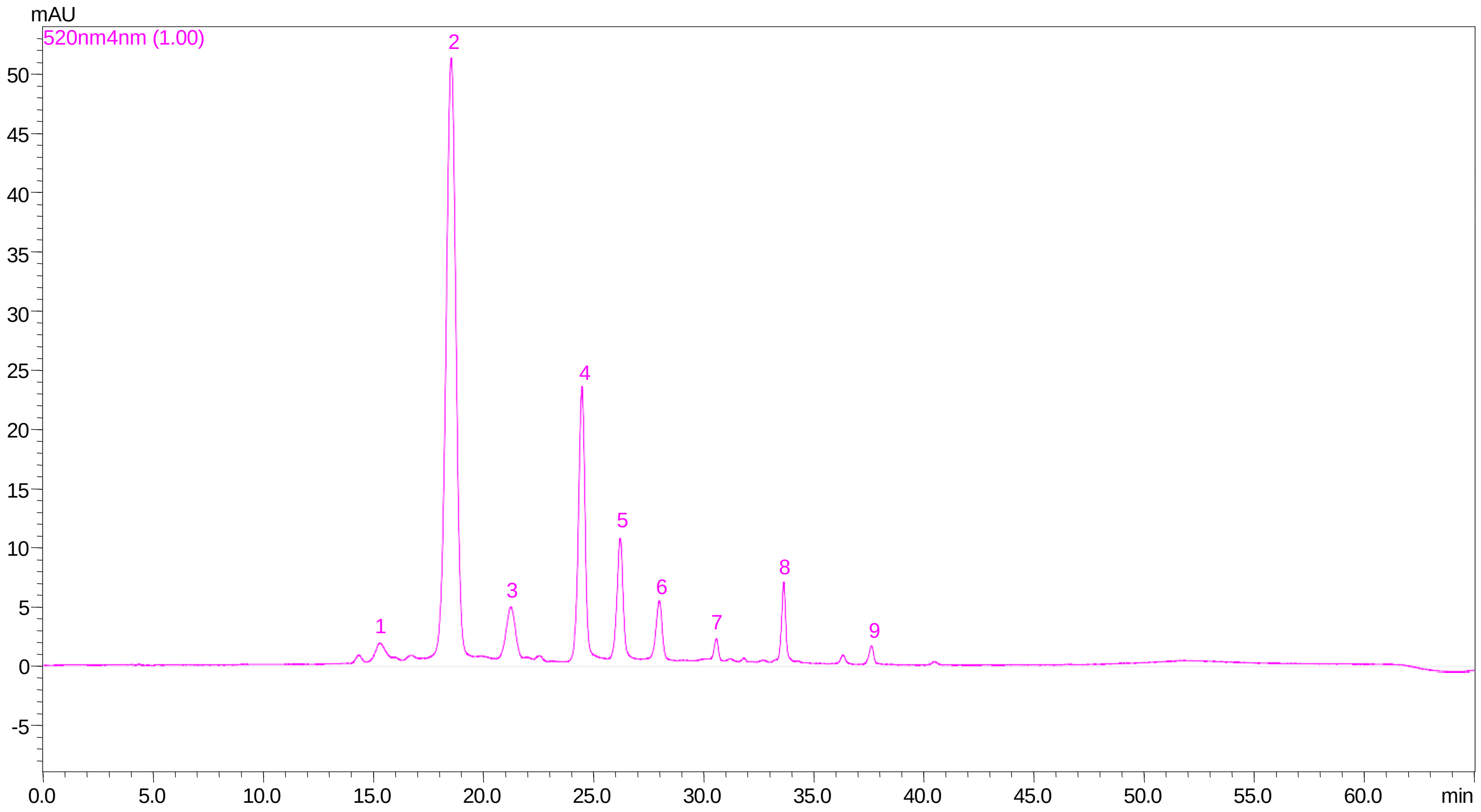


**Figure S16**. The HPLC Chromatogram for anthocyanin separation in **Blake Magic** cultivar

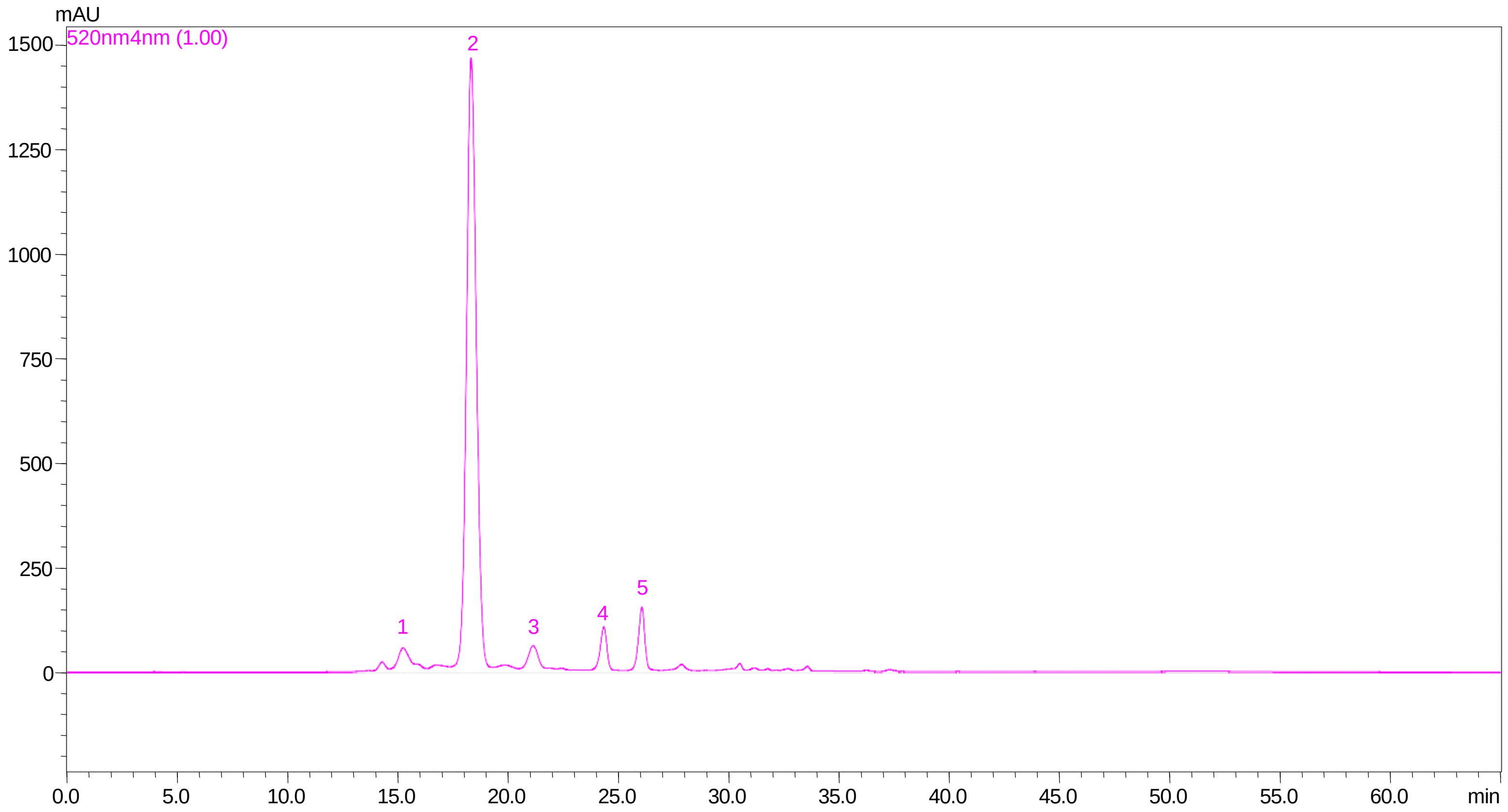


**Figure S17**. The HPLC Chromatogram for anthocyanin separation in **Porta Nigra** cultivar

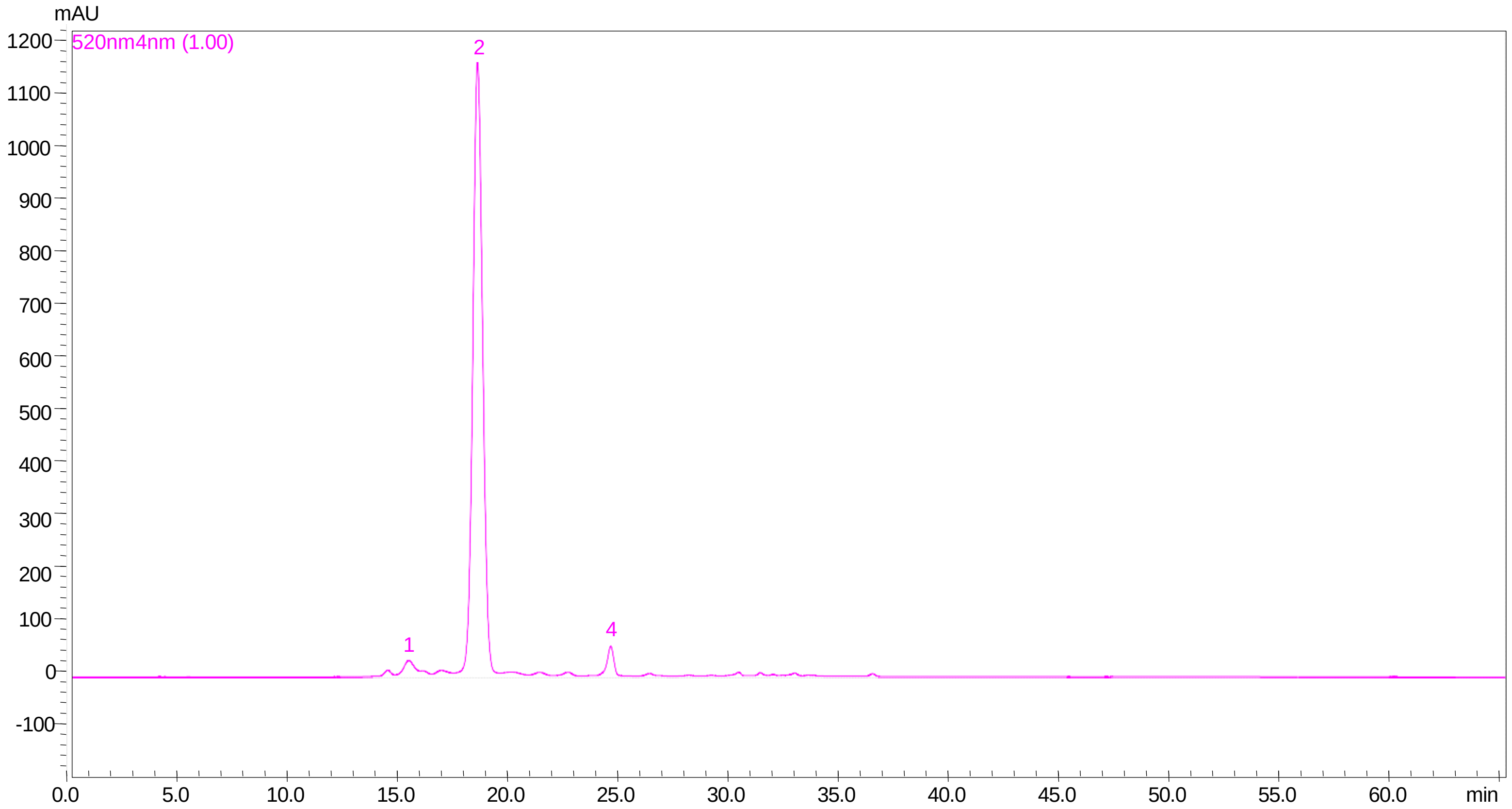


**Figure S18**. The HPLC Chromatogram for anthocyanin separation in **Nina Weibull** cultivar

**Principal component analysis (PCA)**

Principal component analysis (PCA) has emerged as a powerful tool to unravel the intricate patterns of biochemical diversity within the enchanting world of roses. This statistical technique transforms a multitude of interrelated variables, such as the levels of anthocyanins, polyphenols, and flavonoids in rose species, into a smaller set of uncorrelated principal components. These components represent the underlying dimensions of biochemical variation, capturing the most significant trends in the data. By examining the distribution of rose species along these principal components, researchers can identify groups of varieties with similar biochemical profiles. This clustering reveals hidden patterns, providing valuable insights into the biochemical relationships among rose species.

Furthermore, PCA enables researchers to explore the influence of individual biochemical components on the overall variation. By quantifying the contribution of each component to the principal components, researchers can identify the key compounds that drive the biochemical diversity observed in roses. As we delve deeper into the principal component analysis of rose species, we can anticipate the emergence of fascinating insights into the biochemical underpinnings of their diverse characteristics. This knowledge holds immense potential for breeding programs, guiding the development of new rose varieties with enhanced traits, such as vibrant colors, captivating fragrances, and robust resistance to diseases.

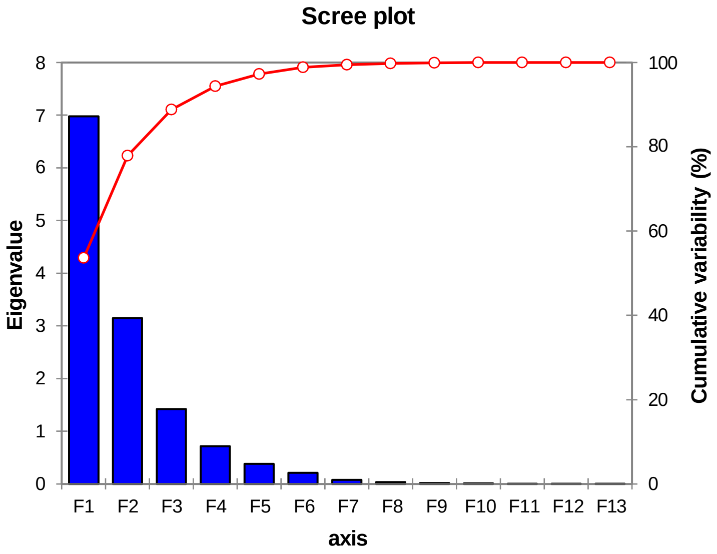


**Figure S19.** Relationship between eigenvalues and principal components.

## Hierarchical clustering

This dendrogram presents the hierarchical clustering of rose varieties based on their total anthocyanin, total polyphenol, total flavonoid content, and individual anthocyanins concentration (Figure S20). The analysis reveals three distinct clusters, with Cluster 1 comprising Cosima and Montana, suggesting a close resemblance in their biochemical profiles. Cluster 2 encompasses a more diverse group, including Orange Triumph, Dame du Coeur, Bunde Liebe, La Sevillana, and additional varieties. Bunde Liebe's presence in both Cluster 2 and 3 might indicate a broader range of shared biochemical characteristics. Cluster 3 groups Olala, Duftzauber, and additional varieties with potentially similar anthocyanin, polyphenol, and flavonoid levels.

While the dendrogram provides valuable insights into the biochemical relationships among these rose varieties, it is crucial to exercise caution when interpreting inter-group influence. The observed clustering doesn't necessarily imply a direct causal relationship between groups. The observed hierarchical clustering in the dendrogram may not necessarily imply a direct influence of Cluster 3 on Clusters 1 and 2. An alternative explanation lies in a shared ancestral origin followed by diversification. Cluster 3 could represent rose varieties that have retained a biochemical profile more closely resembling that of their common ancestor. Conversely, Clusters 1 and 2 might represent lineages that have undergone diversification events due to genetic or environmental pressures, leading to distinct anthocyanin, polyphenol, and flavonoid profiles. This hypothesis of shared ancestry and subsequent diversification can be further investigated through genetic analysis, providing valuable insights into the evolutionary history and biochemical variation within rose varieties.

Further research should delve into the specific biochemical profiles of each variety within their respective clusters, explore the underlying genetic or metabolic mechanisms contributing to the observed groupings, and correlate the biochemical profiles with rose quality and commercial traits like flower color, fragrance, and disease resistance. By addressing these limitations and conducting further research, we can gain a more nuanced understanding of the biochemical relationships among these rose varieties, ultimately informing breeding programs and selection efforts for desired traits.

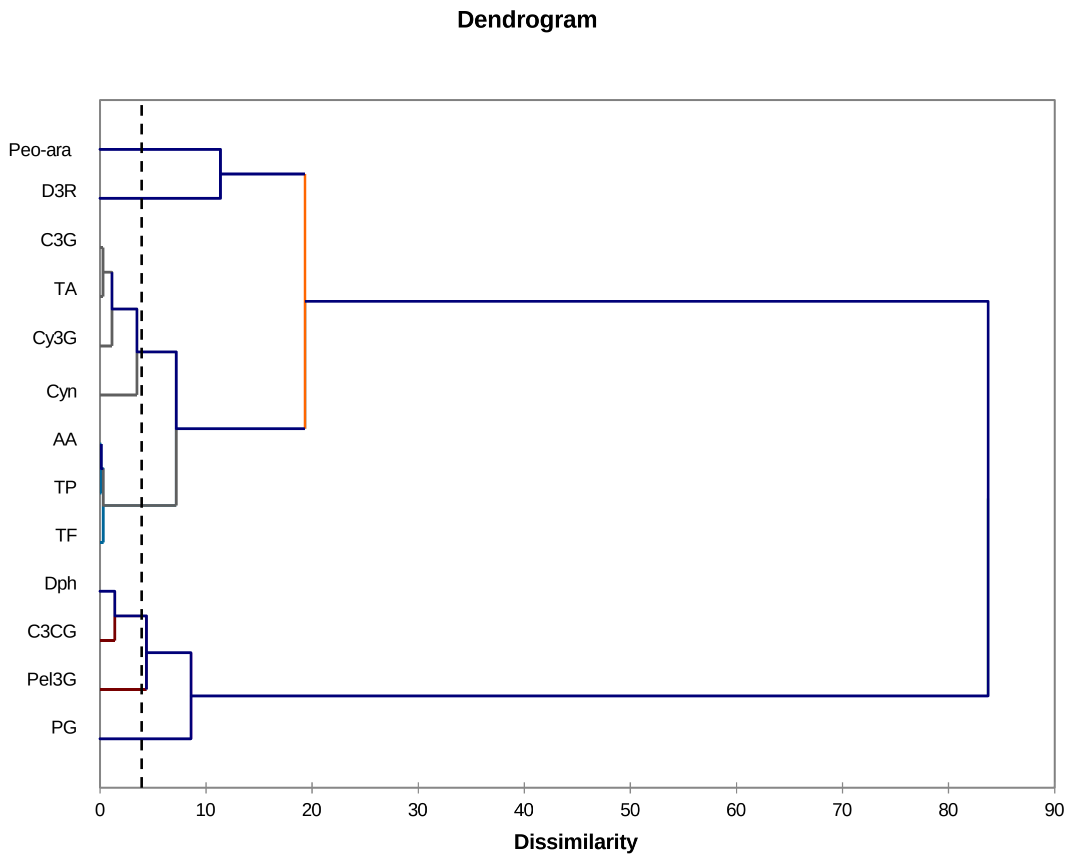


**Figure S20.** Hierarchical clustering of rose varieties based on total anthocyanin, total polyphenol, total flavonoid content, and individual anthocyanins concentration.
